# Buparlisib with thoracic radiotherapy and its effect on tumour hypoxia: A phase I study in patients with advanced non-small cell lung carcinoma

**DOI:** 10.1016/j.ejca.2019.03.015

**Published:** 2019-05

**Authors:** Daniel R. McGowan, Michael Skwarski, Kevin M. Bradley, Leticia Campo, John D. Fenwick, Fergus V. Gleeson, Marcus Green, Amanda Horne, Timothy S. Maughan, Mark G. McCole, Seid Mohammed, Ruth J. Muschel, Stasya M. Ng, Niki Panakis, Remko Prevo, Victoria Y. Strauss, Robert Stuart, Eliana M.C. Tacconi, Katherine A. Vallis, W. Gillies McKenna, Ruth E. Macpherson, Geoff S. Higgins

**Affiliations:** a Department of Oncology, University of Oxford, Oxford, United Kingdom; b Radiation Physics and Protection, Oxford University Hospitals NHS Foundation Trust, Oxford, United Kingdom; c Department of Oncology, Oxford University Hospitals NHS Foundation Trust, Oxford, United Kingdom; d Department of Radiology, Oxford University Hospitals NHS Foundation Trust, Oxford, United Kingdom; e Institute of Translational Medicine, University of Liverpool, Liverpool, United Kingdom; f Department of Cellular Pathology, Oxford University Hospitals NHS Foundation Trust, Oxford, United Kingdom; g Centre for Statistics in Medicine, Nuffield Department of Orthopaedics, Rheumatology and Musculoskeletal Sciences, University of Oxford, Oxford, United Kingdom; h Oncology Clinical Trials Office, Department of Oncology, University of Oxford, Oxford, United Kingdom

**Keywords:** Phase I trial, PI3K inhibitor, NSCLC, Radiotherapy, Tumour hypoxia, FMISO PET-CT

## Abstract

**Background:**

Pre-clinically, phosphoinositide 3-kinase (PI3K) inhibition radiosensitises tumours by increasing intrinsic radiosensitivity and by reducing tumour hypoxia. We assessed whether buparlisib, a class 1 PI3K inhibitor, can be safely combined with radiotherapy in patients with non-small cell lung carcinoma (NSCLC) and investigated its effect on tumour hypoxia.

**Methods:**

This was a 3 + 3 dose escalation and dose expansion phase I trial in patients with advanced NSCLC. Buparlisib dose levels were 50 mg, 80 mg and 100 mg once daily orally for 2 weeks, with palliative thoracic radiotherapy (20 Gy in 5 fractions) delivered during week 2. Tumour hypoxic volume (HV) was measured using ^18^F-fluoromisonidazole positron-emission tomography–computed tomography at baseline and following 1 week of buparlisib.

**Results:**

Twenty-one patients were recruited with 9 patients evaluable for maximum tolerated dose (MTD) analysis. No dose-limiting toxicity was reported; therefore, 100 mg was declared the MTD, and 10 patients received this dose in the expansion phase. Ninety-four percent of treatment-related adverse events were ≤grade 2 with fatigue (67%), nausea (24%) and decreased appetite (19%) most common per patient. One serious adverse event (grade 3 hypoalbuminaemia) was possibly related to buparlisib. No unexpected radiotherapy toxicity was reported. Ten (67%) of 15 patients evaluable for imaging analysis were responders with 20% median reduction in HV at the MTD.

**Conclusion:**

This is the first clinical trial to combine a PI3K inhibitor with radiotherapy in NSCLC and investigate the effects of PI3K inhibition on tumour hypoxia. This combination was well tolerated and PI3K inhibition reduced hypoxia, warranting investigation into whether this novel class of radiosensitisers can improve radiotherapy outcomes.

## Highlights

• Buparlisib, a phosphoinositide 3-kinase (PI3K) inhibitor, is safe when combined with thoracic radiotherapy.
• PI3K inhibition resulted in a rapid reduction in tumour hypoxia in non–small cell lung carcinoma.
• This study supports the development of PI3K inhibitors as novel radiosensitisers.

## 1 Introduction

Radiotherapy is used in half of patients with cancer [1]; however, tumour response and treatment efficacy are highly variable. In locally advanced non-small cell lung carcinoma (NSCLC), outcomes following standard radical (chemo)radiotherapy remain exceptionally poor with 5-year overall survival only in the region of 15% [2]. It is well recognised that suboptimal locoregional control contributes to such poor outcomes [3] and that the development of novel radiosensitisers represents an unmet clinical need for this patient group.

Intracellular signal transduction pathways are known to play an important role in determining tumour response to radiation, thus providing opportunity for developing radiosensitisers [4]. In particular, the well-studied EGFR/Ras/PI3K/Akt pathway appears to be pivotal. Aberrant activation of this pathway is common in many tumour types, including NSCLC, and correlates with poor clinical outcomes after radiotherapy [5], [6], [7]. *In vitro* radiosensitivity studies demonstrate that activation of Ras, PI3K or Akt results in marked resistance of tumour cell lines to radiation, whereas inhibition improves response [8].

The importance of the EGFR/Ras/PI3K/Akt pathway in modifying the tumour microenvironment to alter radiation response has also become apparent, specifically with regard to oxygenation. *In vivo* experiments have shown that inhibitors of EGFR, Ras, PI3K and Akt result in marked ‘normalisation’ of tumour microvasculature with durable increases in perfusion and alleviation of tumour hypoxia [9], [10]. Hypoxic regions are a common feature of solid tumours and result from an imbalance between high oxygen demand and poor oxygen delivery because of dysfunctional tumour vasculature [11]. Hypoxia is associated with an aggressive tumour phenotype and treatment resistance, which is especially pertinent for radiotherapy [12]. There is therefore significant interest in developing hypoxia modifiers as radiosensitisers and the ability of PI3K inhibitors to reduce tumour hypoxia represents a novel class of agents for this purpose. *In vivo* experiments have demonstrated that PI3K inhibition results in significant tumour growth delay after radiation because of vascular remodelling which is independent and synergistic to increasing intrinsic radiosensitivity [13].

Buparlisib (BKM120) (Novartis International AG, Switzerland) is an oral pan class 1 PI3K inhibitor. In xenografts, buparlisib reduces tumour hypoxia through rapid vascular remodelling [13]. *In vitro* studies have also demonstrated that buparlisib inhibits tumour mitochondrial oxygen consumption, thereby further contributing to hypoxia modification [14]. Clinical studies using buparlisib have been conducted in a range of tumour types, with established favourable pharmacokinetics, acceptable toxicity and mixed response rates [15], [16], [17]. Although buparlisib has significant potential to improve radiotherapy response, no previous trials have combined this agent with radiation.

We therefore conducted a phase I clinical trial of buparlisib with thoracic radiotherapy. The primary aim of this study was to investigate the safety and maximum tolerated dose (MTD) of buparlisib in combination with palliative radiotherapy. Palliative radiotherapy was chosen as there were no previous reports of combining this agent with radiation and because of the significant toxicity of radical radiotherapy in NSCLC. This study also investigated the effect of buparlisib on tumour hypoxia, using ^18^F-fluoromisonidazole (FMISO) positron-emission tomography–computed tomography (PET-CT). The use of radiolabelled tracers such as FMISO has become the most widely used method for the clinical study of tumour hypoxia. This non-invasive method correlates with other measures of hypoxia, is highly reproducible and functions as a predictive biomarker of radiotherapy outcomes [18], [19], [20].

Our findings provide clinical evidence for the safety of combining PI3K inhibition with thoracic radiotherapy and for the effect of this class of agents on tumour hypoxia in NSCLC.

## 2 Materials and methods

### 2.1 Study design

This was a single-centre (Oxford Cancer Centre), open-label, dose escalation and expansion phase I clinical trial (EudraCT number: 2012-003762-40). All patients provided written consent and trial conduct complied with the Declaration of Helsinki. Ethical approval was obtained from National Research Ethics Service Committee South Central Oxford B (12/SC/0674).

**Fig. 1:**
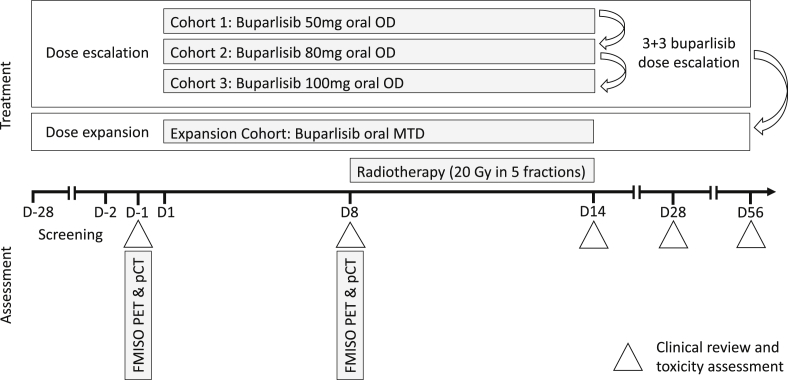
**Trial schema**. D, day; FMISO, ^18^F-fluoromisonidazole; PET, positron-emission tomography; pCT, perfusion computed tomography; OD, once daily; MTD, maximum tolerated dose.

Dose escalation of oral once daily (OD) buparlisib followed a standard 3 + 3 design with three pre-determined dose cohorts: cohort 1 50 mg, cohort 2 80 mg and cohort 3 100 mg. Primary end-points assessed the safety and determined the MTD of buparlisib when combined with palliative thoracic radiotherapy. The MTD was defined as the dose at which no more than 0 of 3 patients or 1 of 6 patients experienced a dose limiting toxicity (DLT). Dose escalation was not permitted beyond 100 mg OD as this dose has previously been established as the single agent MTD [15]. Once the MTD was determined, this dose was used in an expansion cohort of 6 patients with data from all trial patients used to investigate the effect of buparlisib on tumour hypoxia and perfusion after 1 week of treatment (secondary trial end-points). The trial schema is shown in Fig. 1.

### 2.2 Patients

Patients aged ≥18 years with life expectancy of ≥16 weeks, Eastern Cooperative Oncology Group Performance Status of 0–2, histologically confirmed advanced stage NSCLC and a thoracic lesion requiring palliative radiotherapy were eligible. Key exclusion criteria were uncontrolled central nervous system metastases, poorly controlled diabetes mellitus, psychiatric illness, cardiac disease or other malignancy (other than NSCLC) in the last three years. Anti-cancer therapy within 28 days; previous thoracic radiotherapy or exposure to PI3K, mTOR, or Akt inhibitors was not permitted. Full details of the study design including eligibility criteria can be found in the trial protocol provided as Supplementary information.

### 2.3 Treatment regimen

Oral buparlisib was administered OD for 14 days with palliative thoracic radiotherapy delivered during the second week. For radiotherapy planning, patients underwent CT simulation with gross tumour volume outlined and 2 cm margin added for field edge. Treatment was delivered using parallel 6 or 15 MV photon beams, and 20 Gy in 5 daily fractions was prescribed according to International Commission on Radiation Units (ICRU 62) guidance. As palliative radiation was used, no dose constraints were specified.

### 2.4 Assessments

Adverse events (AEs) were graded according to the National Cancer Institute's Common Terminology Criteria (NCI CTCAE version 4.0). DLT was defined as follows: ≥grade 3 non-haematological toxicity (excluding nausea, vomiting or diarrhoea) that required hospitalisation or which did not resolve to ≤grade 2 within 7 days, ≥grade 3 nausea, vomiting or diarrhoea that persisted for >48 h, ≥grade 3 pneumonitis, ≥grade 4 haematological toxicity and grade ≥3 mood change if baseline score was 2 in the self-reported PHQ-9 or GAD-7 mood questionnaire or grade ≥2 mood change if baseline score was ≤1. DLT was considered if toxicity was attributable to buparlisib or its interaction with radiotherapy.

Tumour response was the change in tumour hypoxic volume (HV) as detected by FMISO PET-CT performed at baseline and on day 8 of buparlisib, before radiotherapy. Patients were imaged using a Discovery 690 or 710 PET-CT scanner (GE Healthcare). 370 MBq of FMISO (University of Cambridge, UK) was injected and a 10-min image acquired 4 h after injection. CT was performed for localisation and PET attenuation correction. Tumour outlining was performed by an experienced PET-CT radiologist. Background mean standardised uptake (SUV_mean_) was obtained by outlining blood in the descending aorta. To determine HV, voxel-by-voxel SUVs were divided by the background SUV_mean_ providing tumour-to-blood ratio (TBR) values, and voxels with TBR ≥1.4 were classified hypoxic, as previously described [21]. Volumes of hypoxic voxels before and after buparlisib were compared and ≥10% reduction in HV was defined as a response. This cutoff was based on reproducibility test-retest data for FMISO imaging [20] and used the minimum detectable change (MDC) method [22]. Okamoto *et al.* showed a mean difference of 2.7% and SD 14% in tumour-to-muscle (TMR) volumes from FMISO scans repeated within 48 h (n = 9, excluding two patients with TMR volumes <1.5 mL) [20]. MDC is the smallest change at 95% confidence interval and is defined as the standard error (4.7) multiplied by 1.96, giving 9.2%. Our ≥10% threshold was ratified by the University's independent Early Phase Trial Oversight Committee in combination with an external radiologist.

Changes in tumour perfusion were also investigated using perfusion CT (pCT) imaging. The pCT technique used is provided as Supplementary methodology.

### 2.5 Statistical analysis

Patients were evaluable for DLT analysis after 14 days of buparlisib if they completed 56 days of evaluation or withdrew early after experiencing DLT. Patients who withdrew early for other reasons were deemed non-evaluable and were replaced. All patients who received a dose of buparlisib were included in the safety analysis, for which descriptive statistics were used.

Secondary end-point analysis included all patients who had an interpretable pair of FMISO PET-CT scans. Patients with insufficient baseline HV (<1.5 mL) to reliably measure change were excluded. The number of hypoxia or perfusion responders, median response per cohort and waterfall plots were used to summarise the data. Analysis was undertaken using Stata v15.0 (StataCorp, College Station, TX).

## 3 Results

**Fig. 2:**
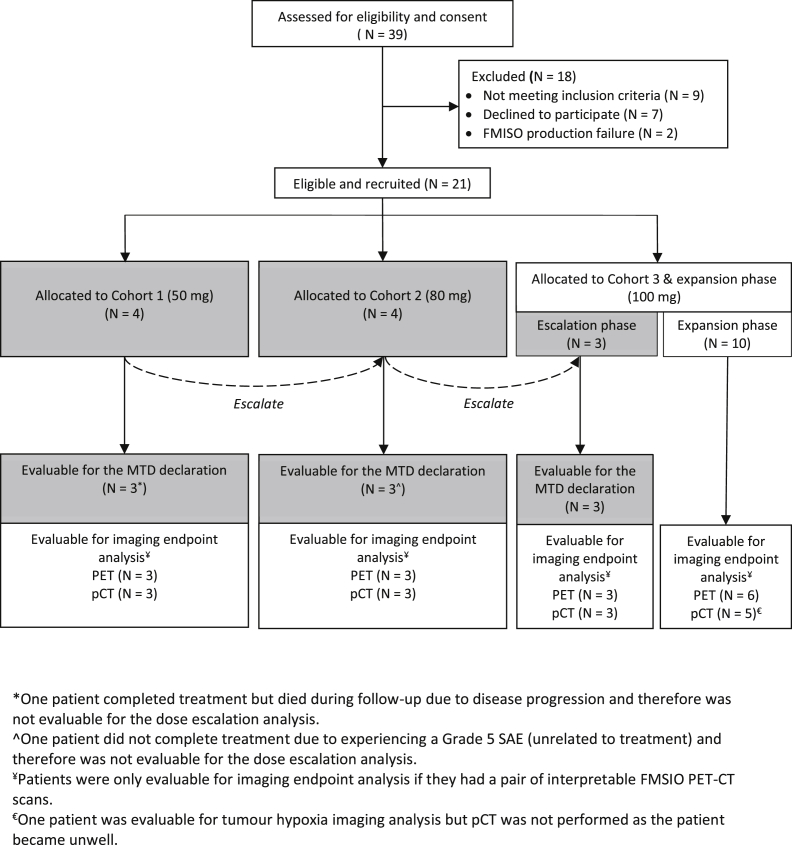
**Consort diagram**. FMISO, ^18^F-fluoromisonidazole; MTD, maximum tolerated dose; PET, positron-emission tomography; pCT, perfusion computed tomography.

From June 2013 to August 2017, 21 patients were recruited. Eleven patients were registered for dose escalation with 9 evaluable for DLT analysis. Ten patients were registered during dose expansion, and in total, 15 patients were evaluable for tumour response analysis. The CONSORT diagram is shown in Fig. 2, and baseline patient characteristics are summarised in Table 1.

**Table 1:** Baseline characteristics.

Characteristics	Dose escalation phase			Expansion phase (n = 10)	Total (n = 21)
	Cohort 1 (n = 4)	Cohort 2 (n = 4)	Cohort 3 (n = 3)		
Age [years]	64 (58–77)	72 (63–75)	68 (68–72)	68 (52–78)	69 (52–78)
Gender					
Male	50 (2)	50 (2)	33 (1)	20 (2)	33 (7)
Female	50 (2)	50 (2)	67 (2)	80 (8)	67 (14)
Stage of disease					
IV	100 (4)	100 (4)	100 (3)	100 (10)	100 (21)
ECOG performance status					
0	0 (0)	0 (0)	33 (1)	50 (5)	29 (6)
1	100 (4)	100 (4)	67 (2)	50 (5)	71 (15)
Tumour volume [mL]^a^	111 (13–510)	135 (29–204)	54 (40–219)	99 (8–250)	101 (8–510)
Histology					
Adenocarcinoma	75 (3)	25 (1)	33 (1)	60 (6)	52 (11)
Squamous cell	25 (1)	75 (3)	67 (2)	40 (4)	48 (10)
Previous treatment					
Chemotherapy	50 (2)	50 (2)	33 (1)	70 (7)	57 (12)
Surgical treatment	0	0	0	60 (6)	29 (6)
Extrathoracic radiotherapy	0	25 (1)	0	30 (3)	19 (4)
Predominant clinical indication for radiotherapy					
Chest pain	25 (1)	0	33 (1)	40 (4)	29 (6)
Bronchial obstruction	75 (3)	25 (1)	0	10 (1)	24 (5)
Cough	0	25 (1)	0	40 (4)	24 (5)
Superior vena cava obstruction	0	25 (1)	0	0	5 (1)
Solitary site of progression	0	25 (1)	0	0	5 (1)
Haemoptysis	0	0	33 (1)	0	5 (1)
Left atrium invasion	0	0	33 (1)	0	5 (1)
Brachial plexus invasion	0	0	0	10 (1)	5 (1)

Data are median (range) or % (number).

ECOG, Eastern Cooperative Oncology Group.

a Tumour volume data were only available for patients evaluable for the imaging analysis.

All 21 patients started buparlisib and 19 patients started radiotherapy. Fifteen (71%) patients had full compliance with treatment. Three patients discontinued treatment because of AEs (described below), 1 patient accidentally missed a dose of buparlisib, 1 patient discontinued treatment because of disease progression and 1 patient was replaced because of FMISO production failure.

### 3.1 MTD and safety assessment

No DLT was reported; therefore, buparlisib 100 mg OD was declared as the MTD. The safety analysis results are summarised in Table 2. In total, 114 AEs were experienced by 20 of 21 patients of which 103 (90%) were ≤grade 2. One patient in the expansion cohort discontinued treatment because of worsening long-standing abdominal pain (unrelated to treatment). 94% of all AEs with any relation to treatment were ≤grade 2 with only three ≥grade 3 AEs deemed possibly related to treatment (2 fatigue and 1 hypoalbuminaemia). Most common treatment-related AEs per patient were fatigue (67%), nausea (24%) and decreased appetite (19%).

**Table 2:** Adverse events.

Parameter	Dose group			Total (n = 21)
	Cohort 1 [50 mg] (n = 4)	Cohort 2 [80 mg] (n = 4)	Cohort 3^a^ [100 mg] (n = 13)	
Total number of AE episodes^b^	19	17	78	114
Patients with				
AEs	4 (100)	4 (100)	12 (92)	20 (95)
Grade ≥ 3 AEs	1 (25)	2 (50)	4 (31)	7 (33)
SAEs	1 (25)	2 (50)	1 (8)	4 (19)
Patients discontinued treatment because of				
AEs	0	0	1 (8)	1 (5)
SAEs	0	1 (25)	1 (8)	2 (10)
Patients experiencing treatment-related AEs^c^				
Fatigue	2 (50)	3 (75)	9 (69)	14 (67)
Nausea	1 (25)	1 (25)	3 (23)	5 (24)
Decreased appetite	1 (25)	0	3 (23)	4 (19)
Constipation	0	0	3 (23)	3 (14)
Radiotherapy skin reaction	1 (25)	0	3 (23)	4 (19)
Rash	0	0	4 (31)	4 (19)
Altered/depressed mood	0	0	3 (23)	3 (14)
Dyspepsia	0	1 (25)	1 (8)	2 (10)
Hiccups	0	0	2 (15)	2 (10)
Oral candidiasis	1 (25)	0	0	1 (5)
Headache	0	1 (25)	0	1 (5)
Weight loss	0	0	1 (8)	1 (5)
Stomatitis	0	0	1 (8)	1 (5)
Personality change	0	0	1 (8)	1 (5)
Dry skin	0	0	1 (8)	1 (5)
Hyperglycaemia	0	0	1 (8)	1 (5)
Hypophosphatemia	0	0	1 (8)	1 (5)
Vomiting	0	0	1 (8)	1 (5)
Cough	0	0	1 (8)	1 (5)
Nightmare	0	0	1 (8)	1 (5)
Total number of treatment-related AEs	6	6	41	53
Patients experiencing treatment-related SAEs				
Hypoalbuminaemia	0	0	1 (8)	1 (5)

Data are patient number (%).

AE, adverse event; SAE, serious adverse event.

a Cohort 3 includes patients in the dose escalation and expansion phases.

b AE episodes are shown only once per patient and if an AE occurrence was temporally associated with study participation, or if the grade of an AE which was present at baseline increased during study participation.

c Shown are AEs of all grades with possible, probable or definite relation to treatment with buparlisib and/or radiotherapy.

Five AEs, all grade 1, were specifically related to radiotherapy and included skin reaction in 3 patients and fatigue and cough in another patient. There was no reported acute oesophagitis or pneumonitis.

Four patients experienced serious adverse events. One patient (cohort 2) discontinued treatment due to grade 5 lower limb ischaemia, which was deemed unrelated to treatment due to long-standing vascular disease and symptoms of acute ischaemia preceded starting buparlisib. One patient (cohort 3) developed grade 3 lung infection (unrelated to treatment) and hypoalbuminaemia (possibly related to buparlisib) and discontinued treatment. Two patients (cohorts 1 and 2) experienced grade 3 lung infection (unrelated to treatment).

### 3.2 Tumour response to buparlisib

**Fig. 3:**
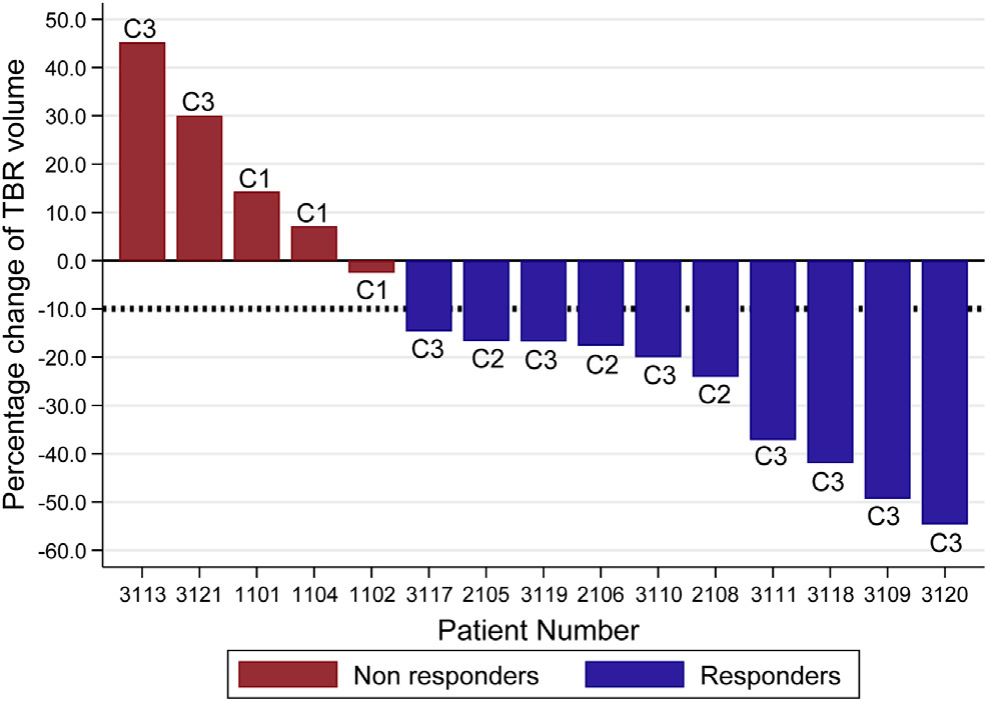
Waterfall plot of change in tumour hypoxic volume. Percentage change of tumour hypoxic volume per patient after 7 days of buparlisib treatment. A ≥10% reduction in hypoxic volume (dotted line) was classified a positive response. C1, cohort 1 (50 mg OD); C2, cohort 2 (80 mg OD); C3, cohort 3 (100 mg OD); FMISO, ^18^F-fluoromisonidazole; TBR, tumour-to-blood FMISO uptake ratio (≥1.4).

**Fig. 4:**
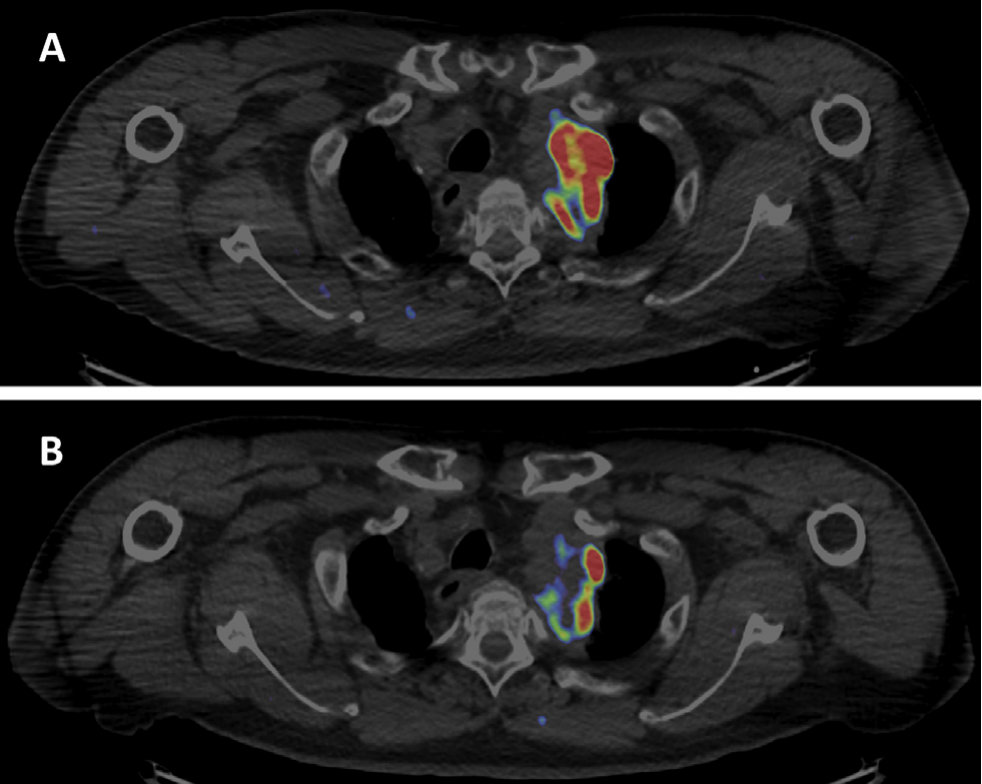
**Example of tumour hypoxic response**. FMISO PET-CT images for one patient in the expansion cohort before (A) and after (B) buparlisib treatment. PET images are fused with the corresponding CT and displayed on a tumour-to-blood uptake ratio (TBR) colour scale. Red regions depict a TBR greater than 1.4, indicating hypoxia, and no visible PET tracer uptake depicts a TBR below 1, indicating normoxia. In this case, there was a 42% reduction in tumour hypoxic volume after buparlisib. FMISO, ^18^F-fluoromisonidazole; PET, positron-emission tomography; CT, computed tomography.

Fifteen patients were evaluable for tumour hypoxia analysis. As shown by the waterfall plot in Fig. 3, 10 (67%) of 15 patients were responders. Table 3 summarises the hypoxia response data. All cohort 1 patients were non-responders with 7% median HV increase. All cohort 2 patients were responders with 18% median HV decrease. In cohort 3 and the expansion cohort, 7 (77%) of 9 patients were responders with 20% median HV decrease. No correlation between tumour size and response was observed (Supplementary Fig. S1). Fig. 4 shows representative examples of FMISO PET-CT images for an expansion cohort patient.

**Table 3:** Summary of FMISO PET-CT results.

Cohort	Number (%) of responders per cohort	TBR >1.4 volume		
		First scan Median [IQR]	Second scan Median [IQR]	% change Median [IQR]
Cohort 1 (n = 3)	0	44.4 [0.4 239]	47.6 [0.4 233]	7.1 [−2.5 14.3]
Cohort 2 (n = 3)	3 (100)	51.3 [1.3 99.5]	42.2 [1.1 75.6]	−17.6 [−24.1 –16.7]
Cohort 3 (n = 9)	7 (77)	33.1 [6.9 43.7]	25.4 [4.9 42.0]	−19.9 [−41.9 –14.6]
**Overall (n = 15)**	**10 (67)**	**40.4 [3.3 67.5]**	**27.6 [3.5 52.5]**	**−16.8 [−37.1 7.1]**

Data are number (%) or median (IQR). Cohort 3 includes patients in the dose escalation and expansion phases.

FMISO, ^18^F-fluoromisonidazole; PET, positron-emission tomography; TBR, tumour-to-blood ratio; IQR, interquartile range.

pCT results are shown as supplementary data (Tables S1 and S2).

## 4 Discussion

We demonstrate that buparlisib, a pan class 1 PI3K inhibitor, is well tolerated with palliative thoracic radiotherapy and that this agent rapidly reduces tumour hypoxia.

Although in our study a palliative dose of radiation was used, the lack of any unexpected radiotherapy toxicity reported is encouraging for the safe combination of this class of agent with radical doses of radiation. Pre-clinical data demonstrate that PI3K inhibition radiosensitises tumours, at least in part, by alleviating tumour hypoxia [13]. As hypoxia is predominantly a tumour-specific phenomenon, in principle, such agents are expected to preferentially radiosensitise tumours, as compared with normal tissues.

Trial accrual was challenging for numerous reasons. Patients with metastatic NSCLC requiring palliative radiotherapy were generally unwell with poor performance status and many were therefore ineligible for the study. Commonly, radiotherapy was indicated urgently and thus trial participation was clinically inappropriate. The high rate of AEs experienced by patients reflects this borderline fit and deteriorating patient group. The increasing use of targeted treatments for advanced NSCLC was a further challenge to recruitment, as was the fact that this was a single-centre study.

Despite the relatively small size of our study, the observation that buparlisib reduces tumour hypoxia supports pre-clinical data and therefore represents an important clinical proof-of-principle for this class of agents. The absence of such studies in the development of hypoxia modifiers previously may explain why numerous clinical trials have failed to demonstrate improvement in radiotherapy outcomes. A lack of sufficiently validated hypoxia biomarkers to enable selection of patients who would benefit from hypoxia treatment is further contributory. For example, in head and neck cancer, the hypoxia-targeting agents nimorazole and tirapazamine may have improved radiotherapy outcomes if predictive hypoxia biomarkers were used to select patients [23], [24]. Therefore, to further develop PI3K inhibitors as radiosensitisers, it is important to also develop and incorporate hypoxia biomarkers into future study design.

As our data demonstrate a reduction in hypoxia after PI3K inhibition, it is hoped that combining such agents with radiotherapy may improve outcomes. To establish this, studies in the radical radiotherapy setting are required. As FMISO PET relies on tracer accumulation in viable tissues with oxygen tensions significantly below that at which radioresistance becomes a feature [25], it is expected that the reduction in HV detected in our study is likely to result in improved tumour radiation response. This is supported by the observation that high FMISO uptake in patients with NSCLC is associated with significantly worse outcomes after radiotherapy [18]. Furthermore, given that PI3K inhibition is known to improve tumour intrinsic radiosensitisation, the effects would be anticipated to be more pronounced than through hypoxia reduction alone. Encouragingly, early phase studies combining inhibitors of downstream signalling targets of PI3K with radiotherapy, such as the AKT inhibitor nelfinavir, have reported promising response rates and outcomes in rectal and pancreatic cancer [26], [27], [28]. Hahn *et al.* demonstrated that upstream inhibition of Ras with a farnesyltransferase inhibitor resulted in impressive complete response rates in NSCLC and head and neck cancer when combined with radical radiotherapy [29].

Although, we observed a reduction in tumour hypoxia in most patients, this did not always correspond with increased tumour perfusion. This may reflect the technical challenges of performing pCT in our patient population, namely tumours were often only partly imaged because of large size with significant motion artefact. Interestingly, this may also perhaps represent the fact that buparlisib inhibits tumour mitochondrial oxygen consumption [14], and so changes in hypoxia and perfusion may, at least in part, be independent phenomena. In fact, mathematical modelling suggests that reducing oxygen consumption may be more effective in addressing tumour hypoxia compared with strategies aimed solely at improving oxygen delivery [30].

## 5 Conclusion

Overall, the results from this trial demonstrate that PI3K inhibition reduces tumour hypoxia in patients with NSCLC and when combined with thoracic radiotherapy is well tolerated. This study supports the development of clinical trials combining this class of agent with radical radiotherapy with the aim of improving outcomes in NSCLC.

## Conflict of interest statement

The authors have no conflicts of interests to declare.
