# Supplementary material for: Buparlisib with thoracic radiotherapy and its effect on tumour hypoxia: A phase I study in patients with advanced non-small cell lung carcinoma
Source: Eur J Cancer. 2019 May;113:87–95. doi: 10.1016/j.ejca.2019.03.015 (PMC6522060; doi:10.1016/j.ejca.2019.03.015)
Supplement: Multimedia component 1 [file mmc1.docx]

**Figure S1**: Graph showing the relationship between the total tumour volume and percentage change in tumour hypoxic volume from baseline (Pearson’s r correlation coefficient, *r* = 0.08).
