# Supplementary material for: Buparlisib with thoracic radiotherapy and its effect on tumour hypoxia: A phase I study in patients with advanced non-small cell lung carcinoma
Source: Eur J Cancer. 2019 May;113:87–95. doi: 10.1016/j.ejca.2019.03.015 (PMC6522060; doi:10.1016/j.ejca.2019.03.015)
Supplement: Multimedia component 2 [file mmc2.docx]

**Supplementary material to: Buparlisib with thoracic radiotherapy and its effect on tumour hypoxia: a phase I study in patients with advanced non-small cell lung carcinoma**

**Supplementary methodology**

*Assessing tumor perfusion using perfusion CT*

For perfusion CT (pCT) patients were imaged supine with their arms raised (if possible) using a Discovery 690 or 710 PET-CT scanner (GE Healthcare, Milwaukee) immediately following the 4-hour post-injection FMISO PET-CT scan. The pCT was limited to a 4 cm region of the tumor axially due to high temporal resolution (one image per second) and CT detector width. For larger tumors this meant that the entire tumor was not fully covered by the pCT scan. Images were acquired in cine mode (120 kV, 60 mA) with 70mL iodinated contrast (Omnipaque 300) injected at 5 mL/s followed by 25 mL water at 5 mL/s. During the 45 second scan the patient was instructed to hold their breath for as long as possible (at inspiration) and to breathe out very slowly if needed in order to limit respiratory motion artefacts. Motion was corrected using software developed by our Engineering Department (1). The motion-corrected pCT data was then processed voxel-by-voxel using the commercial software GE Perfusion 4D (GE Healthcare, Milwaukee, USA) using an input function taken from the mean of a region of interest drawn in the descending aorta to provide blood flow (BF), blood volume (BV) and mean transit time (MTT) parameters. Treatment response was defined as an increase in BF and/or BV and/or a decrease in MTT by ≥25% from baseline, consistent with previously published data (2).

**References**

1. Papież BW, Heinrich MP, Fehrenbach J, Risser L, Schnabel JA. An implicit sliding-motion preserving regularisation via bilateral filtering for deformable image registration. Med Image Anal. 2014;18:1299–311.

2. Sahani DV, Kalva SP, Hamberg LM, Hahn PF, Willett CG, Saini S, et al. Assessing Tumor Perfusion and Treatment Response in Rectal Cancer with Multisection CT: Initial Observations. Radiology. 2005;234:785–92.
