# Supplementary material for: Buparlisib with thoracic radiotherapy and its effect on tumour hypoxia: A phase I study in patients with advanced non-small cell lung carcinoma
Source: Eur J Cancer. 2019 May;113:87–95. doi: 10.1016/j.ejca.2019.03.015 (PMC6522060; doi:10.1016/j.ejca.2019.03.015)
Supplement: Multimedia component 3 [file mmc3.docx]

**Table S1**: Summary of individual patient imaging results

| **Trial number** | **TBR >1.4 volume** | | | **TBR mean** | | | **FMISO-PET Response?** | **pCT BF**  **(mL100g/min)** | | | **pCT BV**  **(mL/100g)** | | | **pCT MTT**  **(s)** | | | **pCT Response?** | **FMISO PET and/or pCT response?** |
| --- | --- | --- | --- | --- | --- | --- | --- | --- | --- | --- | --- | --- | --- | --- | --- | --- | --- | --- |
|  | 1^st^ scan | 2^nd^ scan | % change* | 1^st^ scan | 2^nd^ scan | % change* |  | 1^st^ scan | 2^nd^ scan | % change* | 1^st^ scan | 2^nd^ scan | % change* | 1^st^ scan | 2^nd^ scan | % change* |  |  |
| BKC1101 | 0.4 | 0.4 | 14.3 | 0.89 | 1.04 | 16.82 | NR | 42.0 | 42.6 | 1.4 | 3.6 | 2.6 | -27.8 | 8.6 | 6.2 | -27.9 | R | R |
| BKC1102 | 239.0 | 233.0 | -2.5 | 1.39 | 1.47 | 5.76 | NR | 88.4 | 118.5 | 34.0 | 9.0 | 8.0 | -11.1 | 11.2 | 8.3 | -25.9 | R | R |
| BKC1104 | 44.4 | 47.6 | 7.1 | 1.34 | 1.41 | 5.37 | NR | 64.2 | 60.2 | -6.2 | 4.0 | 3.8 | -5.0 | 6.3 | 5.8 | -7.9 | NR | NR |
| BKC2105 | 1.3 | 1.1 | -16.7 | 0.94 | 0.91 | -3.07 | R | 102.2 | 84.3 | -17.5 | 7.2 | 4.7 | -34.7 | 6.4 | 6.2 | -3.1 | NR | R |
| BKC2106 | 51.3 | 42.2 | -17.6 | 1.20 | 1.07 | -10.73 | R | 55.8 | 36.2 | -35.1 | 4.0 | 3.0 | -25.0 | 8.1 | 10.1 | 24.7 | NR | R |
| BKC2108 | 99.5 | 75.6 | -24.1 | 1.43 | 1.37 | -4.10 | R | 106.2 | 94.5 | -11.0 | 6.5 | 6.2 | -4.6 | 7.8 | 6.1 | -21.8 | NR | R |
| BKC3109 | 7.0 | 3.5 | -49.4 | 1.17 | 1.13 | -3.44 | R | 79.5 | 69.2 | -13.0 | 7.7 | 5.9 | -23.4 | 7.8 | 8.1 | 3.8 | NR | R |
| BKC3110 | 67.5 | 54.0 | -20.0 | 1.23 | 1.17 | -5.54 | R | 69.1 | 35.7 | -48.3 | 7.6 | 3.1 | -59.2 | 10.1 | 9.3 | -7.9 | NR | R |
| BKC3111 | 2.4 | 1.5 | -37.1 | 1.10 | 0.99 | -9.22 | R | 58.6 | 47.4 | -19.1 | 4.8 | 3.2 | -33.3 | 8.2 | 6.9 | -15.9 | NR | R |
| BKC3113 | 3.3 | 4.9 | 45.2 | 1.32 | 1.45 | 10.07 | NR | pCT not performed | | | | | | | | | | NR |
| BKC3117 | 16.7 | 14.2 | -14.6 | 1.22 | 1.21 | -0.73 | R | 176.2 | 146.1 | -17.0 | 7.4 | 6.0 | -19.1 | 5.2 | 4.1 | -19.7 | NR | R |
| BKC3118 | 43.7 | 25.4 | -42.0 | 1.36 | 1.25 | -8.73 | R | 42.2 | 59.8 | 41.7 | 3.7 | 5.4 | 44.6 | 6.0 | 6.9 | 16.5 | R | R |
| BKC3119 | 33.1 | 27.6 | -16.8 | 1.12 | 1.08 | -2.99 | R | 50.6 | 22.9 | -54.7 | 3.6 | 1.3 | -64.6 | 7.5 | 5.1 | -32.5 | R | R |
| BKC3120 | 92.5 | 42.0 | -54.6 | 1.30 | 1.11 | -14.62 | R | 52.3 | 76.3 | 46.0 | 5.0 | 6.2 | 24.5 | 7.4 | 6.3 | -14.7 | R | R |
| BKC3121 | 40.4 | 52.5 | 30.1 | 1.37 | 1.48 | 7.93 | NR | 106.3 | 97.4 | -8.4 | 6.7 | 5.9 | -12.0 | 7.2 | 6.4 | -10.5 | NR | NR |

**% change was calculated using* $\frac{Post result-Pre result}{Pre result} x 100$

Patient BKC3113 was evaluable and a non-responder based on FMISO hypoxia results but pCT was not done due to the patient becoming unwell

Abbreviations: NR, non-responders; R, responders; FMISO, ^18^F-fluoromisonidazole; PET, positron emission tomography; TBR, tumor-to-blood ratio; pCT, perfusion computerised tomography; BF, blood flow; BV, blood volume; MTT, mean transit time
