# Supplementary material for: Buparlisib with thoracic radiotherapy and its effect on tumour hypoxia: A phase I study in patients with advanced non-small cell lung carcinoma
Source: Eur J Cancer. 2019 May;113:87–95. doi: 10.1016/j.ejca.2019.03.015 (PMC6522060; doi:10.1016/j.ejca.2019.03.015)
Supplement: Multimedia component 4 [file mmc4.docx]

**Table S2**: Summary results for perfusion CT

| **Cohort** | **pCT BF (mL/100g/min)** | | | **pCT BV (mL/100g)** | | | **MTT (s)** | | |
| --- | --- | --- | --- | --- | --- | --- | --- | --- | --- |
|  | **First scan**  **Median [IQR]** | **Second scan**  **Median [IQR]** | **% change***  **Median [IQR]** | **First scan**  **Median [IQR]** | **Second scan**  **Median [IQR]** | **% change***  **Median [IQR]** | **First scan**  **Median [IQR]** | **Second scan**  **Median [IQR]** | **% change***  **Median [IQR]** |
| Cohort 1 (n = 3) | 64.2 [42 88.4] | 60.2 [42.6 118.5] | 1.4 [-6.2 34.0] | 4.0 [3.6 9.0] | 3.8 [2.6 8.0] | -11.1 [-27.8 -5.0] | 8.6 [6.3 11.2] | 6.2 [5.8 8.3] | -25.9 [-27.9 -7.9] |
| Cohort 2 (n = 3) | 102.2 [55.8 106.2] | 84.3 [36.2 94.5] | -17.5 [-35.1 -11.0] | 6.5 [4.0 7.2] | 4.7 [3.0 6.2] | -25.0 [34.7 -4.6] | 7.8 [6.4 8.1] | 6.2 [6.1 10.1] | -3.1 [-21.89 24.7] |
| Cohort 3 (n = 8) | 63.9 [51.4 92.9] | 64.5 [41.6 86.9] | -15.0 [-33.7 16.7] | 5.8 [4.3 7.5] | 5.6 [3.2 5.9] | -21.2 [-46.3 6.2] | 7.4 [6.6 8.0] | 6.7 [5.7 7.5] | -12.6 [-17.8 -2.0] |
| Overall (n = 14) | 66.7 [52.3 102.2] | 64.7 [42.6 94.5] | -11.9 [-19.1 1.4] | 5.7 [4.0 7.4] | 5.0 [3.1 5.9] | -21.2 [-33.3 -5.0] | 6.7 [5.7 7.5] | 6.4 [6.1 8.1] | -12.6 [-21.8 -3.1] |

Data are median (IQR). Cohort 3 includes patients in the dose escalation and expansion phases

Abbreviations: pCT, perfusion computerised tomography; TBR, tumor-to-blood ratio; BF, blood flow; BV, blood volume; MTT, mean transit time; IQR, inter-quartile range
