# Supplementary material for: Buparlisib with thoracic radiotherapy and its effect on tumour hypoxia: A phase I study in patients with advanced non-small cell lung carcinoma
Source: Eur J Cancer. 2019 May;113:87–95. doi: 10.1016/j.ejca.2019.03.015 (PMC6522060; doi:10.1016/j.ejca.2019.03.015)
Supplement: Multimedia component 5 [file mmc5.pdf]

BKM120

Full title: A CR-UK phase I study of BKM120 in patients with non-small cell lung cancer (NSCLC) receiving thoracic radiotherapy

Short title: Palliative thoracic radiotherapy plus BKM120

**Protocol Version & date:**	Version 7.0 12Jan2017
**Protocol Number:**	OCTO_031
**Ethics Number:**	12/SC/0674
**EudraCT Number:**	2012-003762-40
**NIHR Portfolio Number:**	13615
**Clintrials.gov. No.**	NCT02128724
**ISRCTN:**	ISRCTN04813360

Sponsored by Oxford University

Confidentiality Statement
This document contains confidential information that must not be disclosed to anyone other than the Sponsor, the Trials Office, the Investigator Team, host NHS Trust(s), regulatory authorities, and members of the Research Ethics Committee.

OCTRU is a UKCRC Registered Clinical Trials Unit
OCTRU is a joint venture between the Centre for Statistics in Medicine (CSM) and the Oncology Clinical Trials Office (OCTO) both based at the University of Oxford

GENERAL CONTACT INFORMATION

**Chief Investigator**	Dr Geoff Higgins<br>Consultant Clinical Oncologist and Clinician Scientist<br>Gray Institute for Radiation Oncology and Biology<br>University of Oxford<br>Old Road Campus Research Building<br>Oxford OX3 7DQ<br>Tel.: 01865 617355<br>Email: geoffrey.higgins@oncology.ox.ac.uk
**Trial Office (OCTO)**	BKM120 Trial Office<br>Oncology Clinical Trials Office (OCTO)<br>Department of Oncology, The University of Oxford<br>Old Road Campus Research Building<br>Oxford OX3 7DQ, UK<br>Tel:+44 (0)1865 617083<br>Email: octo-BKM120@oncology.ox.ac.uk<br>Website: http://www.oncology.ox.ac.uk/trial/bkm120
**Clinical Trial Coordinator (acting)**	Ms Stasya Ng<br>Tel.: +44 (0)1865 617083<br>Fax: +44 (0)1865 617010<br>Email: octo-BKM120@oncology.ox.ac.uk
**Sponsor**	Ms Heather House<br>The University of Oxford Clinical Trials and Research Governance Team<br>Joint Research Office Block 60<br>Churchill Hospital<br>Oxford OX3 7LE<br>Tel: +44 (0)1865 572245<br>Fax: +44 (0)1865 572242<br>E-mail: heather.house@admin.ox.ac.uk
**Lead NHS Trust**	Ms Heather House<br>R&D Department, Oxford University Hospitals NHS Trust<br>Joint Research Department, Block 60, Churchill Hospital<br>Oxford OX3 7LE<br>Tel: +44 (0) 1865 572245<br>E-mail: heather.house@ouh.nhs.uk
**Statistician**	Ms Sharon Love<br>Senior Medical Statistician<br>Oxford Clinical Trials Research Unit (OCTRU)<br>Centre for Statistics in Medicine, The University of Oxford<br>Wolfson College Annexe<br>Linton Road<br>Oxford OX2 6UD<br>Tel : +44 (0)1865 284411<br>Fax: +44 (0)1865 284424<br>Email: sharon.love@csm.ox.ac.uk
	Mr Pradeep Virdee<br>Medical Statistician<br>Centre for Statistics in Medicine<br>University of Oxford<br>Botnar Research Centre<br>Windmill Road

	Oxford OX3 7LD<br>Tel: +44 (0)1865 737950
**Central Laboratory**	BKM120 Sample Handling Laboratory<br>Level 1, Oxford Cancer Centre<br>Old Road<br>Headington<br>Oxford<br>OX3 7LE<br>Tel: +44(0)1865 220557<br>Email: labchurchill@oncology.ox.ac.uk
**Phospho-AKT Expression**	Dr Kevin Myers<br>Department of Oncology, The University of Oxford<br>Old Road Campus Research Building<br>Oxford OX3 7DQ, UK<br>Tel: +44 (0)1865 617050

Clinical Queries

During office hours: Clinical queries should be directed to the BKM120 Trial Office - contact the Clinical Trial Coordinator. The call will be passed on to the Chief Investigator, Clinical Coordinator or an appropriate member of the Trial Management Group.

Out of office hours: call the Churchill Hospital switchboard on Tel: 01865 741 841 and ask to speak to the Clinical Oncology on-call doctor.

Patient Screening: To obtain a screening number please email/fax the Screening Form to the BKM120 Trial Office at octo-BKM120@oncology.ox.ac.uk /01865 617010

Patient Registration: To register a patient on the trial please email/fax the Registration Form to the BKM120 Trial Office at octo-BKM120@oncology.ox.ac.uk/01865 617010

TABLE OF CONTENTS

GENERAL CONTACT INFORMATION 2

PROTOCOL SYNOPSIS 7

SUMMARY SCHEDULE OF EVENTS 12
COHORTS 1-3 PLUS THE EXPANSION COHORT - SCHEDULE OF EVENTS 12
COHORT 4 - SCHEDULE OF EVENTS 13
STUDY FLOW CHART 14

ABBREVIATIONS 15

1 INTRODUCTION 15

1.1 BACKGROUND 15
1.2 OVERVIEW OF BKM120 16
1.3 OTHER RESEARCH INTERVENTIONS 17
1.4 RATIONALE FOR THE STUDY 18

2 TRIAL DESIGN 19

2.1 DURATION OF PATIENT PARTICIPATION 19
2.2 POST-TRIAL CARE AND FOLLOW-UP 19

3 OBJECTIVES AND ENDPOINTS 20

4 PATIENT SELECTION 20

4.1 ELIGIBILITY CRITERIA 20
4.2 PRE-TRIAL SCREENING AND RE-SCREENING (DAY -28 TO DAY -2) 22
4.3 PATIENT REGISTRATION PROCEDURE 22

5 TRIAL ASSESSMENTS AND PROCEDURES 23

5.1 INFORMED CONSENT FOR SCREENING AND MAIN STUDY 23
5.2 PRE-DOSING EVALUATIONS FOR ALL COHORTS 24
5.3 EVALUATIONS DURING THE STUDY FOR COHORTS 1-3 PLUS THE EXPANSION COHORT 24
5.4 EVALUATIONS DURING THE STUDY FOR COHORT 4 26
5.5 END OF STUDY EVALUATIONS 28
5.6 EVALUATIONS ON EARLY WITHDRAWAL 28

6 EARLY PATIENT WITHDRAWAL 28

6.1 PATIENT EVALUABILITY AND REPLACEMENT 28

7 SAMPLES FOR LABORATORY ANALYSIS 29

7.1 SAMPLES TO BE ANALYSED IN LOCAL TRUST'S LABORATORIES 29
7.2 SAMPLES TO BE SENT TO AND ANALYSED IN A CENTRAL LABORATORY 29
7.3 SUMMARY OF RESEARCH SAMPLES TO BE TAKEN DURING THE STUDY 29
7.4 PHARMACODYNAMIC ASSAYS 29
7.5 MOLECULAR STUDIES 29
7.6 LABELLING AND CONFIDENTIALITY OF SAMPLES SENT TO CENTRAL LABORATORIES 29
7.7 CLINICAL REPORTING OF RESEARCH ASSAY RESULTS 29
7.8 TRIAL SAMPLE RETENTION AT END OF STUDY 29
7.9 WITHDRAWAL OF CONSENT FOR SAMPLE COLLECTION AND/OR RETENTION 30

8 INVESTIGATIONAL MEDICINAL PRODUCTS (IMPS) 30

8.1 DOSING SCHEDULES: DOSE LEVELS AND BKM120 DOSE ESCALATION PLAN 30
8.2 MANAGEMENT OF DRUG ADMINISTRATION 31
8.3 SPECIAL PRECAUTIONS 32
8.4 DOSE MODIFICATION 34
8.5 COMPLIANCE 34
8.6 MANAGEMENT OF OVERDOSE 34
8.7 DOSE ESCALATION REVIEW 34

9 OTHER TREATMENTS 34

9.1 BACKGROUND SYSTEMIC THERAPY 34
9.2 SUPPORT MEDICATION 34
9.3 CONCOMITANT MEDICATION AND NON-DRUG THERAPIES 35
9.4 PROHIBITED THERAPIES 35

10 RADIOTHERAPY 36

10.1 RADIOTHERAPY SIMULATION AND PLANNING 36
10.2 RADIOTHERAPY FIELD ARRANGEMENT AND PRESCRIPTION 36
10.3 RADIOTHERAPY TREATMENT DELIVERY 36
10.4 RADIOTHERAPY DOSE CONSTRAINTS 36

11 DRUG MANAGEMENT 37

11.1 DRUG SUPPLIES 37
11.2 DRUG ORDERING 37
11.3 IMP RECEIPT 37
11.4 HANDLING AND STORAGE 37
11.5 LABELLING 37
11.6 DOSING DISPENSING 37
11.7 DRUG ACCOUNTABILITY 37
11.8 DRUG RETURNS FROM PATIENTS 38
11.9 DRUG DESTRUCTION 38
11.10 OCCUPATIONAL SAFETY 38

12 EVALUATION OF RESPONSE TO BKM120 38

12.1 TUMOUR ASSESSMENT 38
12.2 TUMOUR RESPONSE 39
12.3 OTHER DEFINITIONS OF OUTCOME: 39

13 ASSESSMENT OF SAFETY 39

13.1 ADVERSE EVENT DEFINITIONS 39
13.2 CLINICAL LABORATORY ABNORMALITIES AND OTHER ABNORMAL ASSESSMENTS AS AES AND SAES 40
13.3 DETERMINING ADVERSE EVENT CAUSALITY 40
13.4 EXPECTED ADVERSE EVENTS 41
13.5 SUSPECTED UNEXPECTED SERIOUS ADVERSE DRUG REACTIONS (SUSARS) 41
13.6 EXPEDITED REPORTING OF SAES 41
13.7 FOLLOW-UP OF SERIOUS ADVERSE EVENTS 41
13.8 REPORTING ADVERSE EVENTS ON THE CRF 42
13.9 EVENTS EXEMPT FROM BEING REPORTED AS AE/ SAES 42
13.10 INFORMING INVESTIGATORS OF NEW SAFETY INFORMATION 42

14 PREGNANCY 42

15 DEFINING THE END OF TRIAL 43

16 STATISTICAL CONSIDERATIONS 43

16.1 SAMPLE SIZE DETERMINATION 43
16.2 DEFINITION OF PRIMARY AND SECONDARY ENDPOINTS 43
16.3 RANDOMISATION 44

17 STATISTICAL ANALYSIS PLAN 44

17.1 INCLUSION IN ANALYSIS 44
17.2 SUBGROUP ANALYSIS 44
17.3 INTERIM ANALYSES 44
17.4 PROCEDURES FOR REPORTING ANY DEVIATION(S) FROM THE ORIGINAL STATISTICAL PLAN 44
17.5 ANALYSIS OF SAFETY 45
17.6 FINAL ANALYSIS 45

18 TRIAL COMMITTEES 45

18.1 TRIAL MANAGEMENT GROUP (TMG) 45
18.2 DATA AND SAFETY MONITORING 45

18.3 TRIAL STEERING COMMITTEE 45

19 DATA MANAGEMENT 45

19.1 DATABASE CONSIDERATIONS 45
19.2 ELECTRONIC CASE REPORTS FORMS (ECRFS) 46
19.3 ACCOUNTING FOR MISSING, UNUSED, OR SPURIOUS DATA 46

20 CLINICAL STUDY REPORT 46

21 STUDY SITE MANAGEMENT 46

21.1 STUDY SITE RESPONSIBILITIES 46
21.2 STUDY SITE SET UP AND ACTIVATION 46
21.3 STUDY DOCUMENTATION 46

22 REGULATORY AND ETHICAL CONSIDERATIONS 47

22.1 ETHICAL CONDUCT OF THE TRIAL AND ETHICS APPROVAL 47
22.2 REGULATORY AUTHORITY APPROVAL 47
22.3 NHS RESEARCH GOVERNANCE 47
22.4 PROTOCOL AMENDMENTS 47
22.5 URGENT SAFETY MEASURES 47
22.6 TEMPORARY HALT 48
22.7 SERIOUS BREACHES 48
22.8 PROGRESS, END OF STUDY AND OTHER REPORTS 48

23 EXPENSES AND BENEFITS 48

24 QUALITY ASSURANCE 48

24.1 RISK ASSESSMENT AND SITE MONITORING 48
24.2 CENTRAL MONITORING 48
24.3 AUDIT AND REGULATORY INSPECTION 49

25 RECORDS RETENTION & ARCHIVING 49

26 PATIENT CONFIDENTIALITY 49

27 STUDY FUNDING 49

28 SPONSORSHIP AND INDEMNITY 49

28.1 SPONSORSHIP 49
28.2 INDEMNITY 49
28.3 CONTRACTS/AGREEMENTS 50

29 PUBLICATION POLICY 50

30 REFERENCES 50

APPENDIX 1: ECOG PERFORMANCE SCALE 51

APPENDIX 2: LIST OF CYP450 SUBSTRATES TO BE USED WITH CAUTION 52

APPENDIX 3: LIST OF PROHIBITED QT PROLONGING DRUGS 53

APPENDIX 4: LIST OF QT PROLONGING DRUGS TO BE USED WITH CAUTION 53

APPENDIX 5: LIST OF PROHIBITED CYP3A INHIBITORS (STRONG AND MODERATE) AND INDUCERS 54

APPENDIX 6: PATIENT SELF REPORTED MOOD QUESTIONNAIRES 55

APPENDIX 7: AMENDMENT HISTORY 57

PROTOCOL SYNOPSIS

Full Title of study:	A phase I dose escalation study of the PI3K inhibitor BKM120, given concomitantly with palliative radiotherapy for the treatment of Non-Small Cell Lung Cancer (NSCLC).
Short Title:	Palliative thoracic radiotherapy plus BKM120.
Trial Acronym:	BKM120
Objectives:	To determine the safety, dose-limiting toxicity (DLT) and maximum tolerated dose (MTD) of BKM120 when administered concomitantly with thoracic radiotherapy in patients with metastatic NSCLC.
Scientific rationale:	The phosphatidylinositol 3-kinase (PI3K)/Akt/mTOR pathway controls tumour cell proliferation, growth, and survival after DNA damage (4). Activation of this pathway is common in NSCLC and many other cancers and can occur through diverse mechanisms such as amplification of the epidermal growth factor receptor (EGFR) gene, mutations of the Ras oncogene, PI3K mutations and loss of phosphatase and tensin homologue deleted in chromosome 10 (PTEN) (4-7).<br><br>Pre-clinical experiments with BKM120 (PI3K inhibitor) have shown that in addition to inducing tumour cell radiosensitisation *in vitro*, it has a profound effect on the tumour microenvironment and physiology.<br><br>Using xenograft models, it has been shown that BKM120 causes a marked reduction in tumour hypoxia after seven days of treatment. The doses of drug used in these experiments resulted in almost complete reduction of pAKT staining, demonstrating the efficacy of these drugs. Furthermore, power Doppler ultrasound scans showed that seven days of treatment with BKM120 resulted in a significant increase in tumour perfusion.<br><br>The tumour vasculature was significantly altered in animals treated with BKM120 at doses that altered tumour hypoxia and perfusion. These agents induced 'vascular normalisation' by causing the vessels to become longer, wider and less tortuous.<br><br>Importantly it was shown that all of these changes in the tumour microenvironment were associated with marked prolongation in tumour regrowth in the xenografts of animals treated with a combination of radiation and drug treatment compared with treatment with either modality alone.<br><br>Tumours typically have grossly abnormal blood vessels resulting in the tumours becoming hypoxic and poorly perfused. Since poor vascular perfusion and tumour hypoxia significantly reduce the effectiveness of both chemotherapy and radiotherapy respectively, it is possible that treatment with BKM120 may be able to improve the outcomes of NSCLC patients treated with chemotherapy and radiotherapy (8).
Clinical rationale:	BKM120 has previously been used in a single-agent phase I study. It is currently being used in clinical trials in combination with both chemotherapies (e.g. paclitaxel and carboplatin) and biological therapies (trastuzumab). There are currently no trials combining BKM120 with radiotherapy treatment.<br><br>Potential risks to participants include the following:<br>• Exposure to an investigational agent with corresponding side effects. In addition to the main toxicities associated with this treatment (see table in Section 1.4) it is possible that BKM120 may increase the side-effects associated with radiotherapy.<br>• Adding BKM120 to radiotherapy may not be as effective as standard radiotherapy alone. This risk is very small as there is no laboratory evidence to suggest this might happen.<br>• Exposure to ionising radiation from the additional research scans. However, this dose is minimal in comparison with the radiotherapy dose. Additionally,

	the patients in this study have a limited life expectancy due to their advanced cancer and are therefore unlikely to be harmed by the long-term effects of radiation exposure. These scans are purely for research purposes and clinical management is unlikely to be altered as a result of this imaging.<br>• Discomfort and bruising due to the additional research blood tests.<br><br>Potential benefits to participants include:<br>• Access to a novel drug which is not available outside of a clinical trial. BKM120 has shown evidence of clinical activity when used as a single-agent. However, it is not clear whether such responses are likely when the drug is used for shorter periods, as in this trial.<br>• BKM120 may increase the efficacy of radiotherapy. This may result in improved local disease control, with a greater improvement in patient's symptoms.
Primary Endpoint:	Analysis of safety and identification of the maximum tolerated dose (MTD), which is defined as the highest dose at which no more than 1 of 6 evaluable patients or 0 of 3 evaluable patients experience a DLT. DLTs will be defined as per NCI CTCAE v 4.0. The following will be considered DLT if they occur at any point whilst the patient is on study:<br><br>1) Any ≥ grade 3 non-haematological toxicity (excluding nausea, vomiting or diarrhoea) that requires hospital admission or which does not resolve to ≤ grade 2 within 7 consecutive days of optimal treatment.<br>2) Any ≥ grade 3 nausea, vomiting or diarrhoea will be considered DLT only if any of them persist for >48 hours despite maximum supportive care.<br>3) ≥ Grade 3 pneumonitis<br>4) Any ≥ grade 4 haematological toxicity.<br>5) Mood deterioration from baseline (BL). DLT will be any grade ≥3 mood change if BL score of 2. DLT will be any grade ≥2 mood change if BL score of ≤ 1.
Secondary Endpoints:	Changes in ^{18}F-Misonidazole uptake as detected by PET-CT scans. Changes in blood flow as detected by perfusion CT.
Other investigations:	Determine phosphorylation status of Akt in PBMC at baseline, during BKM120 treatment and following BKM120 + RT treatment. Measure tumour pAkt and PTEN levels and then identify mutation status of RAS, PI3K and EGFR by PCR.
Study Design:	Single-centre, open-label, 3+3 cohort, dose escalation phase I study of the use of BKM120 in combination with thoracic radiotherapy.
Patient Numbers:	2-30 evaluable
Target Population:	Patients with incurable NSCLC requiring palliative thoracic radiotherapy.
Inclusion and exclusion criteria	***Inclusion criteria:***<br>A patient will be eligible for inclusion in this study if all of the following criteria apply.<br>1. Evidence of histologically confirmed NSCLC of any stage<br>2. Thoracic lesion requiring palliative radiotherapy and which has been identified on a scan within eight weeks of starting the trial.<br>3. Male or female, age ≥ 18 years at the day of consenting to the study.<br>4. Life expectancy of at least 16 weeks.<br>5. ECOG performance score of 0-2.<br>6. Patient is able to swallow and retain oral medication.<br>7. The patient is willing to provide written informed consent and is likely to comply with the protocol for the duration of the study, and scheduled follow-up visits and examinations.<br>8. Haematological and biochemical indices within the ranges shown below:<br>• Haemoglobin (Hb) ≥ 9.0 g/dL<br>• Absolute neutrophil count ≥ 1.5 x 10^9/L<br>• Platelet count ≥100 x 10^9/L<br>• International Normalised Ratio (INR) ≤ 1.5<br>• Potassium, calcium and Magnesium within normal range<br>• ALT and AST not above normal range or ≤3.0 times ULN if liver metastases

	are present<br>• Total serum bilirubin not above normal range, or ≤1.5 times ULN if liver metastases are present or total bilirubin ≤3.0 times ULN if the patient has well documented Gilbert's disease and absence of other contributing disease process at the time of diagnosis<br>• Creatinine ≤ 1.5 x ULN<br>• Fasting plasma glucose (FPG) ≤ 120mg/dL [6.7 mmol/L]<br><br>***Exclusion criteria:***<br>A patient will **not** be eligible for the trial if **any** of the following apply:<br>1. Previous chemotherapy or biological therapy within four weeks of starting study treatment.<br>2. Treatment with any other investigational agent, or participation in another interventional clinical trial within 28 days prior to enrolment.<br>3. Patient has not recovered to grade 1 or better (except alopecia) from related side effects of any prior antineoplastic therapy.<br>4. Treatment at the start of study treatment with any drugs known to be moderate or strong inhibitors or inducers of isoenzyme CYP3A4, and the treatment cannot be discontinued or switched to a different medication prior to starting study drug.<br>5. Presence of active uncontrolled or symptomatic CNS metastases. Patients with asymptomatic CNS metastases may participate in this trial. Any prior local treatment for CNS metastases must have been completed treatment ≥ 28 days prior to enrolment in the trial (including surgery and radiotherapy).<br>6. Patient has poorly controlled diabetes mellitus (HbA_{1c} > 8 %)<br>7. Previous exposure to PI3K, mTOR, or AKT inhibitor<br>8. Patient has a known hypersensitivity to any of the excipients of BKM120<br>9. Previous thoracic radiotherapy treatment<br>10. Any previous extra-thoracic radiotherapy within 28 days prior to enrolment<br>11. Medically documented history of or active major depressive episode, bipolar disorder, obsessive-compulsive disorder, schizophrenia, a history of suicidal attempt or ideation, or risk of doing harm to others<br>12. Patient meets the cut-off score of ≥ 12 in the PHQ-9 or a cut-off of ≥ 15 in the GAD-7 mood scale, respectively, or selects a positive response of '1, 2, or 3' to question number 9 regarding potential for suicidal thoughts ideation in the PHQ-9 (independent of the total score of the PHQ-9)<br>13. Patient has ≥CTCAE grade 3 anxiety<br>14. Other psychological, social or medical condition, physical examination finding or a laboratory abnormality that the Investigator considers would make the patient a poor trial candidate or could interfere with protocol compliance or the interpretation of trial results.<br>15. Patient has a concurrent malignancy or has had any malignancy (other than NSCLC) in the last 3 years prior to start of study treatment (with the exception of adequately treated basal or squamous cell carcinoma or cervical carcinoma in situ)<br>16. Patient has had major surgery within 14 days of starting the study drug.<br>17. Patient has any other concurrent severe, and/or uncontrolled medical condition that would, in the investigator's judgement contraindicate patient participation in the clinical study (e.g. chronic pancreatitis, chronic active hepatitis).<br>18. Patient has impairment of gastrointestinal (GI) function or GI disease that may significantly alter the absorption of BKM120.<br>19. Patients who are known to be serologically positive for Hepatitis B, Hepatitis C or HIV.<br>20. Patient has active cardiac disease including any of the following:<br>• Left Ventricular Ejection Fraction (LVEF) < 50% as determined by Multiple Gated acquisition (MUGA) scan or echocardiogram (ECHO)<br>• QTc > 480 msec on screening ECG (using the QTcF formula)<br>• Patient is taking a medication that has a known risk of causing QT interval

<table>
<tr><td></td><td>prolongation or inducing Torsades de Pointes, and the treatment cannot be discontinued or switched to an alternative medication.
• Angina pectoris that requires the use of anti-anginal medication
• Ventricular arrhythmias except for benign premature ventricular contractions
• Any other cardiac arrhythmia not controlled with medication
• Supraventricular and nodal arrhythmias requiring a pacemaker or not controlled with medication
• Conduction abnormality requiring a pacemaker
• Valvular disease with documented compromise in cardiac function
• Symptomatic pericarditis
• History of myocardial infarction within 6 months of entering the trial
• History of congestive heart failure(New York Heart Association functional classification III-IV)
• Documented cardiomyopathy
21. Pregnant or breast-feeding women, or women of childbearing potential unless effective methods of contraception are used. Oral contraception, injected or implanted hormonal methods are not allowed as BKM120 potentially decreases the effectiveness of hormonal contraceptives. Acceptable methods of contraception are either:
• True abstinence
• Surgical sterilization
• Male partner sterilization
• Or use of a combination of any two of the following (a+b):
a) Placement of an intrauterine device (IUD) or intrauterine system (IUS)
b) Barrier methods of contraception: condom or occlusive cap (diaphragm or cervical/vault caps) with spermicidal foam/gel/film/cream/vaginal suppository
Women of child-bearing potential must have a negative serum pregnancy test ≤ 72 hours prior to initiating treatment.</td></tr>
<tr><td>Trial dose and administration:</td><td>BKM120 will be administered orally to all cohorts. The trial doses are as below
Cohort 1: 50mg od for a total of two weeks
Cohort 2: 80mg od for a total of two weeks
Cohort 3: 100mg od for a total of two weeks
Expansion cohort: Two weeks of BKM120 at MTD determined by cohorts 1-3.
Cohort 4: Four weeks of BKM120 at the MTD determined by cohorts 1-3.</td></tr>
<tr><td>Radiotherapy schedule</td><td>All patients will receive 20Gy in 5 fractions over one week.</td></tr>
<tr><td>Duration on study:</td><td>Patients in cohorts 1-3 will be on study for 8 weeks.
Patients in cohort 4 will be on study for 10 weeks</td></tr>
<tr><td>Study Procedures and frequency:</td><td>Evaluations at screening day -28 to day -2 (all cohorts)
Written informed consent for Screening and for Main Study, demography, inclusion/exclusion criteria, medical history, diagnosis of cancer, ECOG performance status, patient self-rating mood scales for depression (PHQ-9) and anxiety (GAD-7), ECG, concomitant medications, haematology, biochemistry, research blood sample for PBMC pAkt level, urinalysis.

Evaluations on day -1 (all cohorts)
On the day before the first dose of BKM120 is given:
Physical examination including weight, ECOG performance status, patient self-rating mood scales for depression (PHQ-9) and anxiety (GAD-7), evaluation of toxicity, ^{18}F-Miso PET-CT and perfusion CT scans. Pregnancy test (if applicable). Patients will be supplied with BKM120 to start on day 1.

Evaluations on day 8 and day 14 (cohorts 1-3 and expansion cohort)
Haematology, biochemistry, research blood sample for PBMC pAkt level, physical examination including weight, ECOG performance status, patient self-rating mood scales for depression (PHQ-9) and anxiety (GAD-7), evaluation of toxicity, ^{18}F-Miso PET-CT and perfusion CT scans, urinalysis (day 14 only). Patients will commence radiotherapy treatment on day 8 after the imaging investigations have been completed. Patients will</td></tr>
</table>

	complete both radiotherapy and BKM120 on day 14.<br><br>**Evaluations from day 8 – 28 (cohort 4 only)**<br>Patients will undergo evaluations on an approximately weekly basis whilst receiving BKM120 (at days 8, 15, 22, 28). Haematology, biochemistry, research blood sample for PBMC pAkt level, physical examination including weight, ECOG performance status, patient self-rating mood scales for depression (PHQ-9) and anxiety (GAD-7), evaluation of toxicity. On day 22 patients will also undergo ^{18}F-Miso PET-CT and perfusion CT scans, urinalysis. Commence radiotherapy treatment after the imaging investigations have been completed. Patients will complete treatment on day 28.<br><br>**Evaluations on day 28 (cohorts 1-3 and expansion cohort), day 42 (cohort 4 only)**<br>Two weeks after completion of treatment, patients will undergo:<br>Haematology, biochemistry, research blood sample for PBMC pAkt level, physical examination including weight, ECOG performance status, patient self-rating mood scales for depression (PHQ-9) and anxiety (GAD-7), evaluation of toxicity.<br><br>**Evaluations on day 56 (cohorts 1-3 and expansion cohort), day 70 (cohort 4 only)**<br><br>Six weeks after completion of treatment, patients will undergo:<br>Haematology, biochemistry, research blood sample for PBMC pAkt level, physical examination including weight, ECOG performance status, patient self-rating mood scales for depression (PHQ-9) and anxiety (GAD-7), evaluation of toxicity.<br><br>The end of study visit will occur six weeks after completion of treatment and will involve assessments as described for day 56 (cohorts 1-3 and expansion cohort) or day 70 (cohort 4) above.
Patient care post-trial:	Following the end of study visit, patients will receive standard care. Following discharge to standard clinical care, the responsible healthcare team will be asked to report any clinically significant late radiotherapy effects to the BKM120 Trial Office.
Criteria for evaluation:	
Efficacy:	Response to BKM120 treatment will be based upon changes in tumour hypoxia and perfusion as detected by ^{18}F-Miso PET-CT scans and perfusion CT scans respectively
Safety:	Safety and toxicity will be reported using CTCAE (version 4.0).
Pharmacokinetic assays:	N/A
Pharmacodynamic assays:	The levels of phospho-Akt expression in normal tissues may reflect the efficacy of BKM120. Changes in phospho-Akt expression will be monitored during the trial using PBMCs taken at the timepoints indicated in Sections 5.3 and 5.4. Reductions in phospho-Akt expression of PBMC's following BKM120 treatment will be correlated with changes observed with the functional imaging investigations.
Histopathology:	Histological confirmation of NSCLC
No. of Study Site(s)	Single centre: Oxford, UK
End of study	Last Patient Last Visit. Patients should be on study for a maximum of 10 weeks.
Publication policy	The results of this study will be published in a peer-reviewed scientific journal and presented for other academic research purposes as agreed by the Investigators.

SUMMARY SCHEDULE OF EVENTS
Cohorts 1-3 plus the expansion cohort - Schedule of Events

	Day-28 to day -2	Day -1	Day 1	Day 8	Day 14	Day 28	Day 56
Written Informed Consent Screening	X						
Written Informed Consent Main Study	X						
Demography	X						
Inclusion/exclusion criteria	X						
Relevant medical history/current medical conditions	X						
Diagnosis and extent of cancer	X						
Prior antineoplastic therapy	X						
^{18}F-Miso PET-CT and Perfusion CT scans		X		X			
Blood samples from ^{18}F-Miso PET-CT		X		X			
Vital signs		X		X	X	X	X
Height		X					
Weight		X		X	X	X	X
Physical examination		X		X	X	X	X
ECOG performance status	X	X		X	X	X	X
Patient self-rating mood scales for depression (PHQ-9) and anxiety (GAD-7)	X	X		X	X	X	X
ECG	X				X		X
Prior/concomitant medications	X			X	X	X	X
Adverse Events		X	X	X	X	X	X
Haematology	X			X	X	X	X
Clinical chemistry	X			X	X	X	X
Lipase	X				X		X
Fasting plasma glucose (FPG)	X			X	X	X	X
HbA_{1C}	X						
Liver Function Tests	X			X	X	X	X
Blood test for pAkt measurement	X			X	X	X	X
Coagulation (INR)	X			X	X	X	X
Urinalysis	X				X		X
Dispensing of BKM120		X					
BKM120[a]			Once daily				
Radiotherapy				20 Gy in 5 fractions			
Pregnancy test (if applicable)		X	If clinically indicated				

[a]BKM120 taken at 50mg, 80mg, 100mg (cohorts 1-3 respectively) or MTD (expansion cohort) once daily continuously on days 1-14

Cohort 4 - Schedule of Events

	Day-28 to day -2	Day -1	Day 1	Day 8	Day 15	Day 22	Day 28	Day 42	Day 70
Written Informed Consent Screening	X								
Written Informed Consent Main Study	X								
Demography	X								
Inclusion/exclusion criteria	X								
Relevant medical history/current medical conditions	X								
Diagnosis and extent of cancer	X								
Prior antineoplastic therapy	X								
^{18}F-Miso PET-CT and Perfusion CT scans		X				X			
Blood samples from ^{18}F-Miso PET-CT		X				X			
Vital signs		X		X	X	X	X	X	X
Height		X							
Weight		X		X	X	X	X	X	X
Physical examination		X		X	X	X	X	X	X
ECOG performance status	X	X		X	X	X	X	X	X
Patient self-rating mood scales for depression (PHQ-9) and anxiety (GAD-7)	X	X		X	X	X	X	X	X
ECG	X				X		X		X
Prior/concomitant medications	X			X	X	X	X	X	X
Adverse Events		X	X	X	X	X	X	X	X
Haematology	X			X	X	X	X	X	X
Clinical chemistry	X			X	X	X	X	X	X
Lipase	X				X		X		X
Fasting plasma glucose (FPG)	X			X	X	X	X	X	X
HbA_{1C}	X								
Liver Function Tests	X			X	X	X	X	X	X
Blood test for pAkt measurement	X			X	X	X	X	X	X
Coagulation (INR)	X			X	X	X	X	X	X
Urinalysis	X				X		X		X
Dispensing of BKM120		X							
BKM120[a]			Once daily						
Radiotherapy						20 Gy in 5 fractions			
Pregnancy test (if applicable)		X	If clinically indicated						

[a]BKM120 taken at MTD once daily continuously on days 1-28

Study Flow Chart

Schematic Trial Design of Dose Escalation

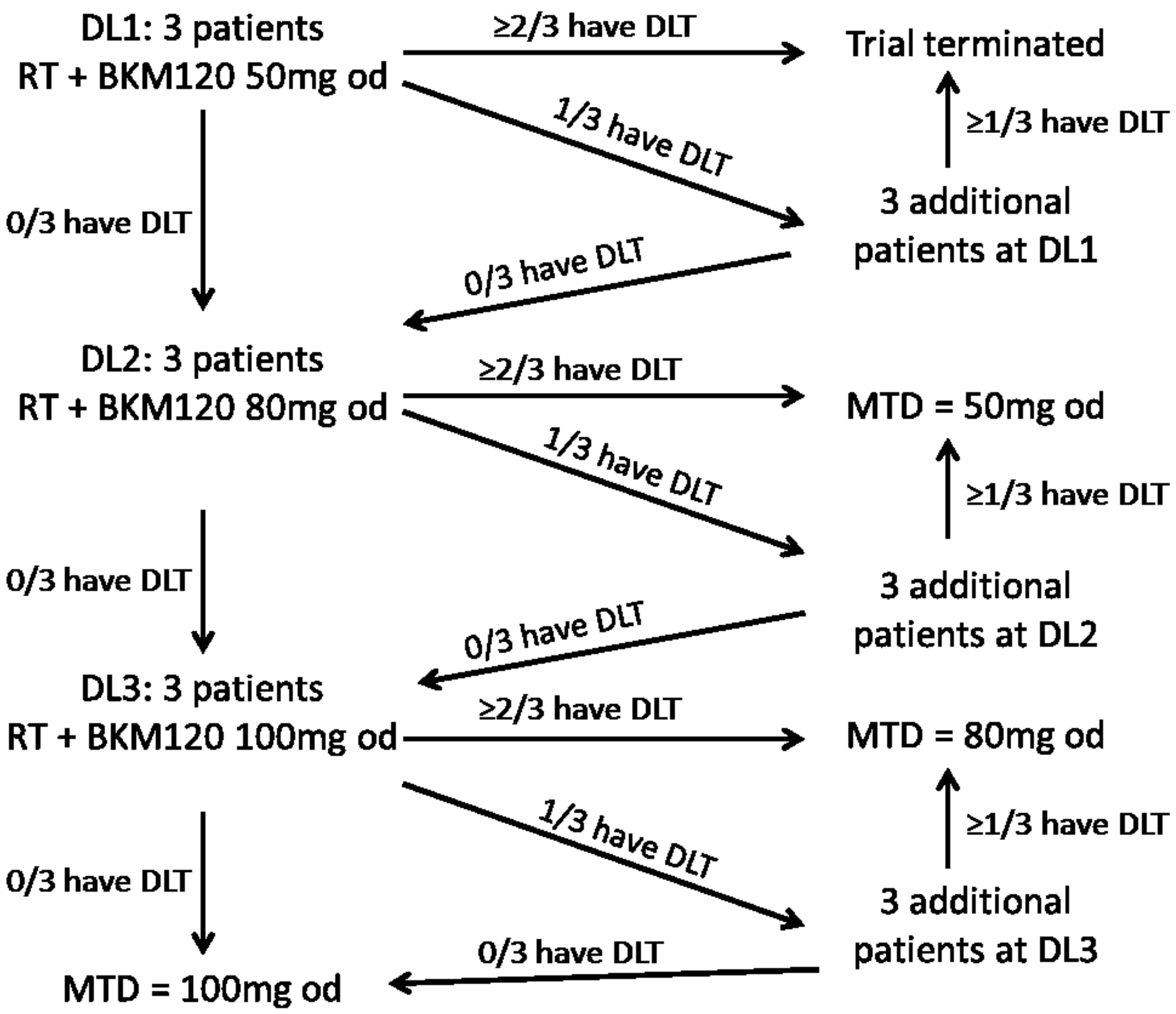

ABBREVIATIONS

AE	Adverse event
CNS	Central Nervous System
CRF	Case report form
CT	Computed tomography
DL	Dose level
DLT	Dose limiting toxicity
eCRF	Electronic case report form
Gy	Gray
FPG	Fasting plasma glucose
FPFV	First patient, first visit
IEPTOC	Independent Early Phase Trial Oversight Committee
ITSC	Independent Trial Steering Committee
IR	Ionising Radiation
LFT	Liver function test
LPLV	Last patient, last visit
MTD	Maximum tolerated dose
NSCLC	Non-small cell lung cancer
OD	Once daily
pAKT	Phosphorlyated AKT
PBMC	Peripheral Blood Mononuclear Cell
PET	Positron emission tomography
PI3K	Phosphatidylinositol 3-kinase
PTEN	Phosphatase and tensin homologue
RPTD	Recommended phase two dose
RT	Radiotherapy
SAE	Serious adverse event
SUSAR	Suspected unexpected serious adverse event
SUV	Standardised uptake value
TMG	Trial management group
ULN	Upper limit of normal

1 INTRODUCTION

1.1 Background

Lung cancer is the most common malignancy in industrialised countries. In 2008 approximately 41000 people in the UK were diagnosed with lung cancer, of which approximately 80% were due to NSCLC. In the same year there were over 35000 deaths due to lung cancer, reflecting the poor prognosis associated with the disease. Radiotherapy plays an important role in the management of patients with NSCLC. Patients with non-metastatic disease may be treated with radical radiotherapy alone (Stage I-II), or with combination radical chemo-radiotherapy treatment (Stage III). Patients with metastatic disease (Stage IV) or with disease not amenable to radical radiotherapy treatment, often receive palliative thoracic radiotherapy in order to alleviate their symptoms.

The overall prognosis associated with NSCLC remains very poor despite improvements associated with combined modality treatment and with technical advances enabling the delivery of more accurate radiotherapy treatment.

Pre-clinical data suggests that BKM120 renders tumour cells more sensitive to ionising radiation and reduces tumour hypoxia and increases perfusion. All of these effects may enhance the efficacy of radiotherapy treatment.

This trial will therefore assess whether BKM120 can be safely combined with thoracic radiotherapy treatment and whether it alters the tumour microenvironment in a way that is likely to improve radiotherapy.

1.2 Overview of BKM120

The investigational agent used in this trial, BKM120 is a potent and highly specific oral, pan class-1 PI3K inhibitor, belonging to the 2,6 dimorpholino pyrimidine derivatives, currently being tested in clinical trials. BKM120 does not have a marketing authorisation. A summary of BKM120 is given below. More detailed information is provided in the BKM120 Investigator's Brochure.

Pharmacodynamics

BKM120 inhibits wild-type PI3Kα (IC50: 35 nM), with at least 50 fold selectivity toward protein kinases (i.e., a panel of cytosolic tyrosine-, cytosolic Serin/Threonin- and receptor tyrosine kinases). The compound is equipotent against somatic PI3Kα mutations (H1047R-, E542K- and E545K-p110α) and is active against the other three PI3K paralogs (PI3Kβ, -γ, -δ; 108 to 348 nM range). BKM120 does not significantly inhibit the related kinases mTor and Vps34, nor does it inhibit (IC50 >10 µM) other receptors and ion channels profiled.

Non-Clinical pharmacokinetics and metabolism

BKM120 showed favourable pharmacokinetic properties in all animal species tested. The absorption of [^{14}C]-BKM120-related radioactivity was >84% in the rat. Oral bioavailability was high in rats (73%), was complete in dogs, and was moderate in monkeys (42%). The estimated steady state plasma volume of distribution (Vss) was high (3.0-3.5L/kg) in all species tested, suggesting a wide tissue distribution. BKM120 was found to cross the blood brain barriers in rats with a tissue-to-plasma ratio of approximately 2 (Novartis internal data). BKM120 is moderately bound to plasma protein in all species examined (about 80%).

In vitro metabolism studies using human liver microsomes showed that oxidative phase I metabolism of BKM120 was predominantly mediated by CYP3A4 (estimated fm>0.9). Formation of a BKM120N-glucoronide conjugate (Phase II metabolism) via the UDP glucuronosyltransferase-1 family, polypeptide A4 (UGT1A4) was also observed in human liver microsomes supplemented with uridine 5'-diphosph-glucuronic acid (UDPGA). BKM120 and metabolites have a low potential for covalent binding to protein.

BKM120 was determined to be a weak reversible inhibitor of CYP3A4 at concentrations reached in patients. BKM120 did not show time-dependent inhibition of CYP450 enzymes. In GLP toxicology studies, BKM120 exposure in terms of AUC0-24 hour and Cmax increased in a dose proportional manner in rat and dog. Results from the rat ADME study showed that radioactivity was mainly excreted into the faeces. Renal excretion was minor. There was no noticeable drug accumulation in dog or male rats after 13 weeks of daily dosing. There was a slight accumulation in female rat (<2 fold).

Non-clinical safety pharmacology and toxicology

Safety pharmacology studies in rats revealed no effects on neuronal (behaviour) or respiratory functions. Cardiac safety studies, conducted in vitro and in vivo did not indicate a prominent electrophysiological risk. No relevant electrophysiological effect was seen in dogs. The only effect considered relevant was a trend toward an increase in systolic and diastolic blood pressure, which was observed in two dog telemetry studies. In rats and dogs, clinical pathology and histopathology findings showed quantitative reductions of lymphoid and erythroid counts and lymphoid tissue hypoplasia.

The pancreas was seen to be affected by treatment with BKM120, particularly in dogs, where acinar cell toxicity was seen in the exocrine part of this organ. At higher doses in the 2-week dose-range-finding study in rats, there were histopathological findings in both the endocrine as well as the exocrine pancreas.

Male sexual organs and associated tissues were found to be targets of toxicity in both rats and dogs. Changes included minimal to slight germ cell depletion, formation of spermatic giant cells and abnormal spermatids, and cellular debris in epididymal tubules. Testicular toxicity did not fully reverse after the 4-week treatment-free period in rats (highest dose), although a clear trend towards recovery was seen. In individual female rats, minimal to slight cyst formation occurred in the Graafian follicles. In dogs, there was no effect on female sexual organs.

Glucose homeostasis was affected in various species (mice, rats, dogs), as expected from the mode of action of BKM120. However, these effects were minimal in both rats and dogs at the doses used in the 4-week studies.

Other safety considerations include:

- After up to 2 weeks of treatment with up to .5mg/kg/day of BKM120, alterations in the levels of multiple brain neurotransmitters were seen in rats.
- No evidence for a direct DNA interaction was found in an Ames test and two chromosome aberration tests in vitro with BKM120. However, evidence of a genotoxic potential with BKM120 has been seen in vitro and in vivo and is likely due to an aneugenic effect.
- No phototoxic potential or any effect on wound healing has been identified with BKM120 in pre-clinical studies.

In conclusion, pre-clinical data found that the majority of observed effects were related to the pharmacological activity of BKM120 as an inhibitor of PI3K, such as a potential influence on glucose homeostasis and the risk of increased blood pressure.

Pharmacodynamic biomarkers

The preclinical in vivo studies with xenografted tumours in mice indicate that detectable inhibition of AKT phosphorylation, which is an accurate readout of PI3K activity, as well as further suppression of downstream signaling (eg phosphorylation of S6) was obtained soon after BKM120 administration.

Clinical pharmacokinetics

Preliminary review of the available pharmacokinetic data showed that BKM120 is rapidly absorbed after oral administration with mean peak plasma concentrations (C_{max}) obtained between 0.5 to 4 h post dose (t_{max}). The median t_{max} at the MTD dose (100 mg daily) was about 1 hour. After reaching C_{max}, BKM120 plasma concentrations decreased in a bi-exponential manner. At the MTD dose (100 mg daily), mean C_{max} and drug exposure within a dosing interval (AUC0-24) was ~1600 ng/ml (CV=45%) and ~ 20,000 ng.hr/ml (CV=40%), respectively. Apparent total body clearance from plasma (CL/F) was low: ~5.0 L/h, indicating that BKM120 is a low clearance drug. BKM120 accumulated ~3-fold in achieving steady state, consistent with an effective half-life of ~40 h. Steady-state can be expected to be reached after approximately 7-10 days of daily dosing in most patients. Approximate dose proportional increase in C_{max} and AUC was found in the dose range of 12.5-150 mg. Intersubject variability in C_{max} and AUC differed at each dose level but was relatively low and generally around 40%.

Clinical experience with BKM120

As of September 2012, over 500 patients have been entered into clinical studies with BKM120. Overall the treatment has been well tolerated. The most common adverse events associated with BKM120 are anorexia, nausea, constipation, diarrhoea, fatigue, rash, hyperglycaemia, asthenia, abdominal pain, vomiting, liver toxicity, anxiety and depression. Potential adverse events requiring special precaution are mentioned in Section 8.3.

It has previously been recommended that the recommended phase two dose (RPTD) for single agent BKM120 should be 100mg once daily (od) (1). Since it is conceivable that BKM120 may increase the side-effects associated with radiotherapy treatment, the initial cohorts of patients will be treated with lower doses of BKM120. The first cohort will be treated with 50mg od, escalating to 80mg od and then 100mg od for subsequent cohorts if no safety issues emerge.

The first three cohorts of patients will be treated with BKM120 for one week prior to commencing radiotherapy treatment. This is based on pre-clinical experiments which showed that one week of BKM120 treatment significantly reduced tumour hypoxia and increased tumour perfusion. Since it may take longer in humans to affect the microenvironment in this way, an optional cohort of patients will receive 3 weeks of BKM120 prior to radiotherapy (cohort 4) if no functional imaging changes are detected in the one week pre-treatment cohorts.

1.3 Other research interventions

This trial will use functional imaging investigations to identify changes in tumour physiology caused by BKM120. ^{18}F-Misonidazole (^{18}F-Miso) PET-CT scans and perfusion CT scans will be used to measure tumour hypoxia and perfusion respectively. They will be conducted prior to commencing BKM120 and repeated prior to commencing radiotherapy treatment.

^{18}F-Miso is a radiotracer that selectively accumulates in hypoxic tissues. ^{18}F-Miso PET-CT scans are therefore able to non-invasively image tumour hypoxia and have been shown to be capable of detecting changes in tumour hypoxia due to therapeutic drug treatment (2).

Perfusion CT is a technique used to study the vasculature within tumours and other tissues (3). It is non-invasive and readily incorporated into existing CT protocols using conventional contrast agents.

If BKM120 increases the efficacy of radiotherapy treatment, it is likely that it will do so largely by reversing tumour hypoxia and increasing tumour perfusion. These functional imaging investigations are therefore extremely important in determining whether BKM120 causes these changes in tumour physiology and at what doses these changes are apparent.

1.4 Rationale for the study

Non-clinical

The phosphatidylinositol 3-kinase (PI3K)/Akt/mTOR pathway controls tumour cell proliferation, growth, and survival after DNA damage (4). Activation of this pathway is common in NSCLC and many other cancers and can occur through diverse mechanisms such as amplification of the epidermal growth factor receptor (EGFR) gene, mutations of the Ras oncogene, PI3K mutations and loss of phosphatase and tensin homologue deleted in chromosome 10 (PTEN) (4-7).

Pre-clinical experiments with BKM120 have shown that in addition to inducing tumour cell radiosensitisation *in vitro*, they have a profound effect on the tumour microenvironment and physiology.

Using xenograft models, it has been shown that BKM120 causes a marked reduction in tumour hypoxia after seven days of treatment. The doses of drugs used in these experiments resulted in almost complete reduction of pAKT staining, demonstrating the efficacy of these drugs. Furthermore, power Doppler ultrasound scans showed that seven days of treatment with BKM120 resulted in a significant increase in tumour perfusion.

The tumour vasculature was significantly altered in animals treated with BKM120 at doses that altered tumour hypoxia and perfusion. These agents induced 'vascular normalisation' by causing the vessels to become longer, wider and less tortuous.

Importantly it was shown that all of these changes in the tumour microenvironment were associated with marked prolongation in tumour regrowth in the xenografts of animals treated with a combination of radiation and drug treatment compared with treatment with either modality alone.

Tumours typically have grossly abnormal blood vessels resulting in the tumours becoming hypoxic and poorly perfused. Since poor vascular perfusion and tumour hypoxia significantly reduce the effectiveness of both chemotherapy and radiotherapy respectively, it is possible that treatment with BKM120 may be able to improve the outcomes of NSCLC patients treated with chemotherapy and radiotherapy (8).

Clinical

BKM120 has previously been used in a single-agent phase I study (summarised in the table below). It is currently being used in clinical trials in combination with both chemotherapies (e.g. paclitaxel and carboplatin) and biological therapies (trastuzumab). There are currently no trials combining BKM120 with radiotherapy treatment.

Potential risks to participants include the following:

- Exposure to an investigational agent with corresponding side effects. In addition to the main toxicities associated with this treatment (see table below) it is possible that BKM120 may increase the side-effects associated with radiotherapy.
- Adding BKM120 to radiotherapy may not be as effective as standard radiotherapy alone. This risk is very small as there is no laboratory evidence to suggest this might happen.
- Exposure to ionising radiation from the additional research scans. However, this dose is minimal in comparison with the radiotherapy dose. Additionally, the patients in this study have a limited life expectancy due to their advanced cancer and are therefore unlikely to be harmed by the long-term effects of radiation

exposure. These scans are purely for research purposes and clinical management is unlikely to be altered as a result of this imaging.
- Discomfort and bruising due to the additional research blood tests.

Potential benefits to participants include:
- Access to a novel drug which is not available outside of a clinical trial. BKM120 has shown evidence of clinical activity when used as a single-agent. However, it is not clear whether such responses are likely when the drug is used for shorter periods, as in this trial.
- BKM120 may increase the efficacy of radiotherapy. This may result in improved local disease control, with a greater improvement in patient’s symptoms.

Table of relevant clinical studies

Trial name/ researcher/ Pub. Ref.	Phase	Disease type	No. of patients	Main Toxicities	Outcome
Bendell et al (1)	I	All advanced solid tumours	35	Hyperglycaemia, upper abdominal pain, rash, mood alteration, nausea	MTD of BKM120 established (100mg od)

2 TRIAL DESIGN

This study will be a single-centre, open-label, 3+3 cohort, dose escalation phase I study of the use of BKM120 in combination with thoracic radiotherapy. Patients with incurable NSCLC requiring palliative thoracic radiotherapy will be eligible for entry. Potential patients will be initially screened to determine eligibility for the main study. For the main study, the first three cohorts of patients will be treated with escalating doses of BKM120. These patients will be treated with BKM120 for a total of fourteen days. One week after commencing BKM120, patients will start palliative radiotherapy treatment. Radiotherapy treatment will be delivered as 20Gy in 5 fractions over a one week period. The delay for radiotherapy, compared to not participating in the trial, for cohorts 1-3 and the expansion cohort will only be a few days and the delay for cohort 4 will be approximately 2 weeks. However since the effects of radiotherapy typically take several weeks to show any benefit the delays are unlikely to change the effect of the radiotherapy.

The MTD will be determined as described in Section 8.1. An additional expansion cohort of a further six patients will be treated with BKM120 in combination with radiotherapy at whichever cohort (1-3) corresponds to the MTD.

In the event that no changes are detected in tumour hypoxia or perfusion in cohorts 1-3, an optional group of patients (cohort 4) will be recruited. These patients will receive BKM120 treatment for a total of 28 days. Three weeks after commencing BKM120, this cohort will receive palliative radiotherapy with 20Gy in 5 fractions over a one week period.

The trial will recruit 2-30 evaluable patients presenting with any stage of NSCLC, requiring palliative radiotherapy treatment.

Refer to the schedule of events and flow chart for details of the study visits and procedures.

2.1 Duration of patient participation

Participants in cohorts 1-3 and the expansion cohort will be in the study for approximately 8 weeks, and participants in cohort 4, will be in the study for approximately 10 weeks from the start of BKM120 treatment.

2.2 Post-trial care and follow-up

Following the end of study visit, patients will receive standard care. Following discharge to standard clinical care, the responsible healthcare team will be asked to report any clinically significant late radiotherapy effects to the trial office for up to 1 year following the end of treatment.

3 OBJECTIVES AND ENDPOINTS

Primary Objective	Endpoints/ Outcome measures
• To determine the safety, dose-limiting toxicity (DLT) and MTD of BKM120 when administered concomitantly with thoracic radiotherapy in patients with incurable NSCLC	Analysis of safety and find the MTD of BKM120.<br>DLTs will be defined per NCI CTCAE v 4.0. The following will be considered DLT if they occur at any point whilst the patient is on study:<br>1) Any ≥ grade 3 non-haematological toxicity (excluding nausea, vomiting or diarrhoea) that requires hospital admission or which does not resolve to ≤ grade 2 within 7 consecutive days of optimal treatment.<br>2) Any ≥ grade 3 nausea, vomiting or diarrhoea will be considered DLT only if any of them persist for >48 hours despite maximum supportive care.<br>3) ≥ Grade 3 pneumonitis<br>4) Any ≥ grade 4 haematological toxicity.<br>5) Mood deterioration from baseline. DLT will be any grade ≥3 mood change if BL score of 2. DLT will be any grade ≥2 mood change if BL score of ≤ 1.The maximum tolerated dose (MTD) is defined as the highest dose at which no more than 1 of 6 evaluable patients or 0 of 3 evaluable patients experience a DLT (see section 6.1).
**Secondary Objectives**	**Endpoints**
• To investigate whether BKM120 alters tumour hypoxia and perfusion	• Changes in ^{18}F-Misonidazole uptake as detected by PET-CT scans.<br>• Changes in blood flow as detected by perfusion CT.
**Tertiary/Exploratory Objectives**	**Endpoints**
• To evaluate Akt phosphorylation as a predictive marker of response to BKM120	• Determine phosphorylation status of Akt in PBMC at baseline, during BKM120 treatment and following BKM120 + RT treatment.
• To investigate potential biomarkers that correlate with response to BKM120	• Measure tumour pAkt and PTEN levels and then identify mutation status of RAS, PI3K and EGFR by PCR.

4 PATIENT SELECTION

All patients will be screened for inclusion and exclusion criteria within 4 weeks prior to the first dose of BKM120. Baseline evaluations should be performed within 4 weeks of the first dose of BKM120. See Section 5.2 for a description of the baseline evaluations required. Written informed consent must be obtained before any study specific procedures are performed. The Investigator will determine patient eligibility based on the following criteria.

4.1 Eligibility criteria

Inclusion criteria:

A patient will be eligible for inclusion in this study if all of the following criteria apply.

1. Evidence of histologically confirmed NSCLC of any stage
2. Thoracic lesion requiring palliative radiotherapy and which has been identified on a scan within eight weeks of starting the trial.
3. Male or female, age ≥ 18 years at the day of consenting to the study.
4. Life expectancy of at least 16 weeks.
5. ECOG performance score of 0-2.
6. Patient is able to swallow and retain oral medication.

7. The patient is willing to provide written informed consent and is likely to comply with the protocol for the duration of the study, and scheduled follow-up visits and examinations.
8. Haematological and biochemical indices within the ranges shown below:

Lab Test	Value required
Haemoglobin (Hb)	≥ 9.0 g/dL
Absolute neutrophil count	≥ 1.5 x 10^9/L
Platelet count	≥100 x 10^9/L
International Normalised Ratio (INR)	≤ 1.5
Potassium, calcium and Magnesium	Within normal range
ALT and AST	Not above normal range or ≤3.0 times ULN if liver metastases are present
Total serum bilirubin	Not above normal range, or ≤1.5 times ULN if liver metastases are present or total bilirubin ≤3.0 times ULN if the chief investigator is satisfied that the patient has well documented Gilbert's disease and absence of other contributing disease process at the time of diagnosis
Creatinine	≤ 1.5 x ULN
Fasting plasma glucose (FPG)	≤ 120mg/dL [6.7 mmol/L]

Exclusion criteria:
A patient will not be eligible for the trial if **any** of the following apply:

1. Previous chemotherapy or biological therapy within four weeks of starting study treatment.
2. Treatment with any other investigational agent, or participation in another interventional clinical trial within 28 days prior to enrolment.
3. Patient has not recovered to grade 1 or better (except alopecia) from related side effects of any prior antineoplastic therapy.
4. Treatment at the start of study treatment with any drugs known to be moderate or strong inhibitors or inducers of isoenzyme CYP3A4, and the treatment cannot be discontinued or switched to a different medication prior to starting study drug.
5. Presence of active uncontrolled or symptomatic CNS metastases. Patients with asymptomatic CNS metastases may participate in this trial. Any prior local treatment for CNS metastases must have been completed treatment ≥ 28 days prior to enrolment in the trial (including surgery and radiotherapy).
6. Patient has poorly controlled diabetes mellitus (HbA_{1c} > 8 %)
7. Previous exposure to PI3K, mTOR, or AKT inhibitor
8. Patient has a known hypersensitivity to any of the excipients of BKM120
9. Previous thoracic radiotherapy treatment
10. Any previous extra-thoracic radiotherapy within 28 days prior to enrolment
11. Medically documented history of or active major depressive episode, bipolar disorder, obsessive-compulsive disorder, schizophrenia, a history of suicidal attempt or ideation, or risk of doing harm to others
12. Patient meets the cut-off score of ≥ 12 in the PHQ-9 or a cut-off of ≥ 15 in the GAD-7 mood scale, respectively, or selects a positive response of '1, 2, or 3' to question number 9 regarding potential for suicidal thoughts ideation in the PHQ-9 (independent of the total score of the PHQ-9)
13. Patient has ≥CTCAE grade 3 anxiety
14. Other psychological, social or medical condition, physical examination finding or a laboratory abnormality that the Investigator considers would make the patient a poor trial candidate or could interfere with protocol compliance or the interpretation of trial results.
15. Patient has a concurrent malignancy or has had any malignancy (other than NSCLC) in the last 3 years prior to start of study treatment (with the exception of adequately treated basal or squamous cell carcinoma or cervical carcinoma in situ)
16. Patient has had major surgery within 14 days of starting the study drug.
17. Patient has any other concurrent severe, and/or uncontrolled medical condition that would, in the investigator's judgement contraindicate patient participation in the clinical study (e.g. chronic pancreatitis, chronic active hepatitis).
18. Patient has impairment of gastrointestinal (GI) function or GI disease that may significantly alter the absorption of BKM120.

19. Patients who are known to be serologically positive for Hepatitis B, Hepatitis C or HIV.
20. Patient has active cardiac disease including any of the following:
    - Left Ventricular Ejection Fraction (LVEF) < 50% as determined by Multiple Gated acquisition (MUGA) scan or echocardiogram (ECHO)
    - QTc > 480 msec on screening ECG (using the QTcF formula)
    - Patient is taking a medication that has a known risk of causing QT interval prolongation or inducing Torsades de Pointes, and the treatment cannot be discontinued or switched to an alternative medication.
    - Angina pectoris that requires the use of anti-anginal medication
    - Ventricular arrhythmias except for benign premature ventricular contractions
    - Any other cardiac arrhythmia not controlled with medication
    - Supraventricular and nodal arrhythmias requiring a pacemaker or not controlled with medication
    - Conduction abnormality requiring a pacemaker
    - Valvular disease with documented compromise in cardiac function
    - Symptomatic pericarditis
    - History of myocardial infarction within 6 months of entering the trial
    - History of congestive heart failure(New York Heart Association functional classification III-IV)
    - Documented cardiomyopathy
21. Pregnant or breast-feeding women, or women of childbearing potential unless effective methods of contraception are used. Women of child-bearing potential, defined as all women physiologically capable of becoming pregnant, must use highly effective methods of contraception. Oral contraception, injected or implanted hormonal methods are not allowed as BKM120 potentially decreases the effectiveness of hormonal contraceptives. Acceptable methods of contraception are either:
    - True abstinence: When this is in line with the preferred and usual lifestyle of the patient. [Periodic abstinence (eg calendar, ovulation, sympotothermal, post ovulation methods) and withdrawal are not acceptable methods of contraception].
    - Surgical sterilization: Have had bilateral oophorectomy (with or without hysterectomy) or tubal ligation at least six weeks before taking BKM120. In case of oophorectomy alone, only when the reproductive status of the woman has been confirmed by follow up hormone level assessment.
    - Male partner sterilization (with the appropriate post-vasectomy documentation of the absence of sperm in the ejaculate). [For female study patients, the vasectomised male partner should be the sole partner for that patient]
    - Or use of a combination of any two of the following (a+b):

    a) Placement of an intrauterine device (IUD) or intrauterine system (IUS)
    b) Barrier methods of contraception: condom or occlusive cap (diaphragm or cervical/vault caps) with spermicidal foam/gel/film/cream/vaginal suppository

    Female patients must use acceptable methods of contraception must continue to use contraception for at least 4 weeks after completing BKM120. Male patients (and their female partners) will need to continue to use contraception for at least 16 weeks after completing BKM120.

    Women of child-bearing potential must have a negative serum pregnancy test ≤ 72 hours prior to initiating treatment

4.2 Pre-trial screening and re-screening (day -28 to day -2)

Patients will be eligible for screening provided that:

- They give separate written informed consent for completing the self-rating mood questionnaires.
- The Investigator anticipates that there is a reasonable expectation that they will satisfy the remaining inclusion criteria see section 4.1 above (NB formal screening for the main study should not be performed until the outcome of the mood questionnaires of the patient is known).

To obtain a screening number, complete a Screening Form and fax/email it to the BKM120 Trial Office.

Patients who do not meet the inclusion/exclusion criteria first time round will not be re-screened.

4.3 Patient registration procedure

Potential participants will have been referred to oncology services for management of NSCLC and be considered suitable for palliative radiotherapy treatment by their multidisciplinary team. The study team will maintain screening

and study enrolment logs for all patients approached concerning the study. The log will include details of patients subsequently excluded along with the reason for the exclusion.

Protocol waivers to deviate from the entry criteria will not be granted.

A patient registration/eligibility check-list must be completed and passed to the BKM120 Trial Office.

Before entering a patient onto the study the Principal Investigator or designee will confirm eligibility. If in any doubt the Chief Investigator must be consulted before entering the patient. Details of the query and outcome of the decision must be documented on the registration/eligibility checklist.

After completing suitability checks, the Informed Consent Form for trial participation and the Registration Form for the patient, site staff will email/fax the Registration Form to the BKM120 Trial Office to confirm the patient's eligibility. The patient will then be registered, where applicable, to one of the cohorts.
Once the investigator / research nurse has a study number for the patient, they will be asked to provide the original Registration Form and a copy of the patient's histology report (which will identify the patient by study number only) to the Trials office. This will allow the Trial Office to confirm the disease stage of patients entering the study.

5 TRIAL ASSESSMENTS AND PROCEDURES

Please refer to the Schedule of Events given at the front of this protocol. Details of all protocol evaluations and investigations must be recorded in the patient's medical record for extraction onto the CRF.

5.1 Informed consent for screening and main study

Informed consent will be a two stage process. Firstly potential participants will be asked to provide consent for screening. If the potential patients are successfully screened they will be asked to consent to the main study.

Potential participants will be given a current, approved version of the patient information sheet. They will also receive clear verbal information about the study detailing no less than: the nature of the study; the implications and constraints of the protocol; the known side effects and any risks involved in taking part. It will be explained that they will be free to withdraw from the study at any time, for any reason, without prejudice to future care, and with no obligation to give a reason for withdrawal.

They will have at least 24 hours to consider the information provided and the opportunity to question the Investigator, their GP or other independent parties before deciding whether to participate. The Investigator or designee who obtains consent must be suitably qualified and experienced, and be authorised to do so by the Chief/Principal Investigator. The Investigator is responsible for ensuring that the trial consent procedures comply with current applicable GCP Regulatory and ethical requirements. Informed consent discussions and outcomes must be well documented in the medical record. The Investigator or designee must be satisfied that the patient has made an informed decision before taking consent. The patient and the Investigator or designee must personally sign and date the current approved version of the informed consent form in each other's presence. A copy of the information and signed consent form will be given to the participant. The original signed form will be retained at the study site. A copy must be held in the Investigator Site File if retaining the original in the medical record.

Contraceptive/ Pregnancy counselling
All participants must be advised on the need to use reliable methods of contraception during the study. The advice should include:

(1) The acceptable methods, including: male or female sterilization, some intrauterine devices (IUDs), and abstinence.
(2) The recommendation that a barrier method should be used in addition to another form of contraception.
(3) Males should continue to take these precautions for a minimum of 16 weeks after the last dose of study drug. Female partners of men enrolled in the study should continue to use acceptable methods of contraception for 16 weeks after the final dose of BKM120.
(4) Female patients in the study should continue to take these precautions for a minimum of 4 weeks after the last dose of study drug.

(5) That any pregnancy that occurs during administration of the study drug will be followed up and the outcome of all pregnancies (spontaneous miscarriage, elective termination, normal birth or congenital abnormality) will be reported and followed up even if the participant is discontinued from the study.

Evaluations at screening (day -28 to day -2) for all cohorts

- Written informed consent for Screening.
- Patient self-rating mood scales for depression (PHQ-9) and anxiety (GAD-7).

5.2 Pre-dosing evaluations for all cohorts

The following must be performed/obtained within 4 weeks before the patient receives the first study dose.

- Written informed consent for Main Study.
- Demographic details.
- Medical History including prior diagnosis, prior treatment and concomitant diseases.
- Concomitant medications.
- ECOG performance status.
- Patient self-rating mood scales for depression (PHQ-9) and anxiety (GAD-7).
- Haematology – Hb, white blood cells (WBC) with differential count (neutrophils and lymphocytes), platelets and INR.
- Biochemistry – sodium, potassium, calcium, magnesium, phosphate, urea, creatinine, albumin, bilirubin, alkaline phosphatase (alk phos), AST, ALT, LDH, HbA1c and lipase.
- Fasting plasma glucose (FPG) ≤ 120mg/dL [6.7 mmol/L].
- Research blood sample for PBMC pAkt level.
- Urinalysis (Blood, White Cells, Protein and Nitrites) &/or dipstick for proteinuria.
- Electrocardiogram (ECG).
- Histological confirmation of NSCLC.

5.3 Evaluations during the study for cohorts 1-3 plus the expansion cohort

An illustration of the schedule for patients in cohorts 1-3 is shown below. Treatment with BKM120 starts on Day 1 and continues until day 14. Radiotherapy treatment starts on day 8 and continues until day 14.

The MTD will be determined as described in Section 8.1. An additional expansion cohort of a further six patients will be treated with BKM120 in combination with radiotherapy at whichever cohort (1-3) corresponds to the MTD.

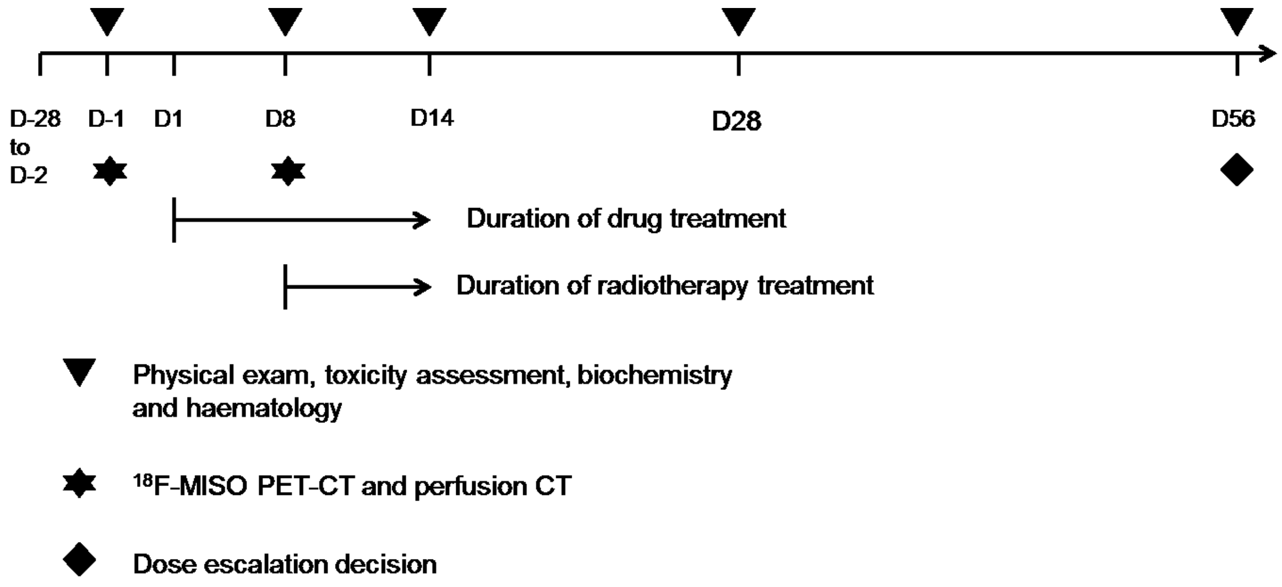

The breakdown of the investigations performed at each visit for patients in cohorts 1-3 are summarised below:

Evaluations on day -1

On the day **before** the first dose of BKM120 is given:

- Physical examination including height and weight.
- ECOG performance status.
- Patient self-rating mood scales for depression (PHQ-9) and anxiety (GAD-7).
- Vital signs: systolic / diastolic blood pressure (BP), pulse rate.
- Evaluation of toxicity (NCI CTCAE v 4.0).
- Patients will undergo ^{18}F-Miso PET-CT and perfusion CT scans.
- Blood samples from ^{18}F-Miso PET-CT.
- Pregnancy test: serum or urine Human Chorionic Gonadotropin (HCG) test to rule out pregnancy at study entry; results must be obtained and reviewed before the first dose of BKM120 is administered.
- Patients will be supplied with BKM120 and will commence treatment on day 1.

Evaluations on day 8

- Haematology – Hb, white blood cells (WBC) with differential count (neutrophils and lymphocytes), platelets and INR.
- Biochemistry – sodium, potassium, calcium, magnesium, phosphate, urea, creatinine, albumin, bilirubin, alkaline phosphatase (alk phos), AST, ALT and LDH.
- FPG.
- Research blood sample for PBMC pAkt level.
- Physical examination including weight.
- Concomitant medications.
- ECOG performance status.
- Patient self-rating mood scales for depression (PHQ-9) and anxiety (GAD-7).
- Vital signs: systolic / diastolic blood pressure (BP), pulse rate.
- Evaluation of toxicity (NCI CTCAE v 4.0).
- Patients will undergo ^{18}F-Miso PET-CT and perfusion CT scans.
- Blood samples from ^{18}F-Miso PET-CT.
- Patients will commence radiotherapy treatment after the imaging investigations have been completed.

Evaluations on day 14

- Haematology – Hb, white blood cells (WBC) with differential count (neutrophils and lymphocytes), platelets and INR.
- Biochemistry – sodium, potassium, calcium, magnesium, phosphate, urea, creatinine, albumin, bilirubin, alkaline phosphatase (alk phos), AST, ALT, LDH and lipase.
- FPG.
- Urinalysis (Blood, White Cells, Protein and Nitrites) &/or dipstick for proteinuria.
- Electrocardiogram (ECG).
- Research blood sample for PBMC pAkt level.
- Physical examination including weight.
- Concomitant medications.
- ECOG performance status.
- Patient self-rating mood scales for depression (PHQ-9) and anxiety (GAD-7).
- Vital signs: systolic / diastolic blood pressure (BP), pulse rate.
- Evaluation of toxicity (NCI CTCAE v 4.0).
- Patients will complete both radiotherapy and BKM120.

Evaluations on day 28

Two weeks after completion of treatment, patients will undergo:

- Haematology – Hb, white blood cells (WBC) with differential count (neutrophils and lymphocytes), platelets and INR.
- Biochemistry – sodium, potassium, calcium, magnesium, phosphate, urea, creatinine, albumin, bilirubin, alkaline phosphatase (alk phos), AST, ALT and LDH.
- FPG.

- Research blood sample for PBMC pAkt level.
- Physical examination including weight.
- Concomitant medications.
- ECOG performance status.
- Patient self-rating mood scales for depression (PHQ-9) and anxiety (GAD-7).
- Vital signs: systolic / diastolic blood pressure (BP), pulse rate.
- Evaluation of toxicity (NCI CTCAE v 4.0).

Evaluations on day 56

Six weeks after completion of treatment, patients will undergo:

- Haematology – Hb, white blood cells (WBC) with differential count (neutrophils and lymphocytes), platelets and INR.
- Biochemistry – sodium, potassium, calcium, magnesium, phosphate, urea, creatinine, albumin, bilirubin, alkaline phosphatase (alk phos), AST, ALT, LDH and lipase.
- FPG.
- Urinalysis (Blood, White Cells, Protein and Nitrites) &/or dipstick for proteinuria.
- Electrocardiogram (ECG).
- Research blood sample for PBMC pAkt level.
- Physical examination including weight.
- Concomitant medications.
- ECOG performance status.
- Patient self-rating mood scales for depression (PHQ-9) and anxiety (GAD-7).
- Vital signs: systolic / diastolic blood pressure (BP), pulse rate.
- Evaluation of toxicity (NCI CTCAE v 4.0).

A dose escalation decision will be made after the last patient in each cohort has completed day 56 evaluation. The dose escalation will be based on the prevalence of dose limiting toxicities as defined in Section 8.1.

5.4 Evaluations during the study for cohort 4

An illustration of the schedule for patients in cohorts 4 is shown below. Treatment with BKM120 starts on Day 1 and continues until day 28. Radiotherapy treatment starts on day 22 and continues until day 28.

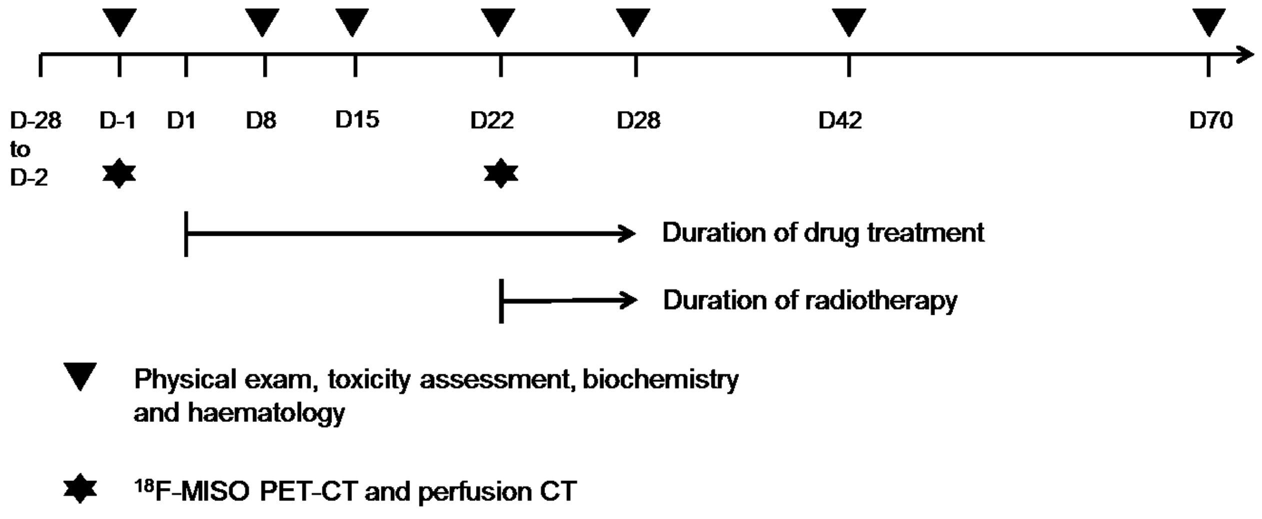

The breakdown of the investigations performed at each visit for patients in cohort 4 are summarised below:

Evaluations on day -1
On the day **before** the first dose of BKM120 is given
- Physical examination including height and weight.
- ECOG performance status.
- Patient self-rating mood scales for depression (PHQ-9) and anxiety (GAD-7).
- Vital signs: systolic / diastolic blood pressure (BP), pulse rate.
- Evaluation of toxicity (NCI CTCAE v 4.0).
- Patients will undergo ^{18}F-Miso PET-CT and perfusion CT scans.
- Blood samples from ^{18}F-Miso PET-CT.
- Pregnancy test: serum or urine Human Chorionic Gonadotropin (HCG) test to rule out pregnancy at study entry; results must be obtained and reviewed before the first dose of BKM120 is administered.
- Patients will be supplied with BKM120 and will commence treatment on day 1.

Evaluations from day 8 – 28
Patients will undergo evaluations on an approximately weekly basis whilst receiving BKM120 (at days 8, 15, 22, 28).
- Haematology – Hb, white blood cells (WBC) with differential count (neutrophils and lymphocytes), platelets and INR.
- Biochemistry – sodium, potassium, calcium, magnesium, phosphate, urea, creatinine, albumin, bilirubin, alkaline phosphatase (alk phos), AST, ALT and LDH. Lipase (days 15 and 28 only).
- FPG.
- Urinalysis (Blood, White Cells, Protein and Nitrites) &/or dipstick for proteinuria (days 15 and 28 only).
- Electrocardiogram (ECG) (days 15 and 28 only).
- Research blood sample for PBMC pAkt level.
- Physical examination including weight.
- Concomitant medications.
- ECOG performance status.
- Patient self-rating mood scales for depression (PHQ-9) and anxiety (GAD-7).
- Vital signs: systolic / diastolic blood pressure (BP), pulse rate.
- Evaluation of toxicity (NCI CTCAE v 4.0).
- Patients will complete treatment on day 37.

On **day 22** patients will also:
- Undergo ^{18}F-Miso PET-CT and perfusion CT scans.
- Blood samples from ^{18}F-Miso PET-CT.
- Commence radiotherapy treatment after the imaging investigations have been completed.

Evaluations on day 42
Two weeks after completion of treatment, patients will undergo:
- Haematology – Hb, white blood cells (WBC) with differential count (neutrophils and lymphocytes), platelets and INR.
- Biochemistry – sodium, potassium, calcium, magnesium, phosphate, urea, creatinine, albumin, bilirubin, alkaline phosphatase (alk phos), AST, ALT and LDH.
- FPG.
- Research blood sample for PBMC pAkt level.
- Physical examination including weight.
- Concomitant medications.
- ECOG performance status.
- Patient self-rating mood scales for depression (PHQ-9) and anxiety (GAD-7).
- Vital signs: systolic / diastolic blood pressure (BP), pulse rate.
- Evaluation of toxicity (NCI CTCAE v 4.0).

Evaluations on day 70
Six weeks after completion of treatment, patients will undergo:
- Haematology – Hb, white blood cells (WBC) with differential count (neutrophils and lymphocytes), platelets and INR.
- Biochemistry – sodium, potassium, calcium, magnesium, phosphate, urea, creatinine, albumin, bilirubin, alkaline phosphatase (alk phos), AST, ALT, LDH and lipase.

- FPG.
- Urinalysis (Blood, White Cells, Protein and Nitrites) &/or dipstick for proteinuria.
- Electrocardiogram (ECG).
- Research blood sample for PBMC pAkt level.
- Physical examination including weight.
- Concomitant medications.
- ECOG performance status.
- Patient self-rating mood scales for depression (PHQ-9) and anxiety (GAD-7).
- Vital signs: systolic / diastolic blood pressure (BP), pulse rate.
- Evaluation of toxicity (NCI CTCAE v 4.0).

5.5 End of study evaluations

The end of study visit will occur six weeks after completion of treatment and will involve assessments as described in Sections 5.3 and 5.4.

5.6 Evaluations on early withdrawal

Where possible, patients who withdraw early from the study will be evaluated as described in the 'End of study evaluations' section above.

6 EARLY PATIENT WITHDRAWAL

Treatment Withdrawal
During the course of the trial, a patient may withdraw early from treatment. This may happen for a number of reasons, including:

Unacceptable toxicity
AE/SAEs requiring discontinuation
Clinical decision
Patient decision

When the patient stops treatment early, the End of Study Form needs to be completed, along with any other relevant CRFs (e.g. a SAE Form). The reason for withdrawing early from treatment should be clearly documented in the medical records.

Consent Withdrawal
Consent withdrawal means that a patient has expressed a wish to withdraw from the study altogether. Under these circumstances, the site needs to document all relevant discussions in the patient notes and notify the Trials Office, which will allow the office to mark all future CRFs as not applicable.

Under these conditions, investigators are still responsible to follow up any SAEs till resolution.

6.1 Patient evaluability and replacement

Dose escalation
The MTD will be based on DLTs observed within 6 weeks of completion of BKM120 treatment. DLTs will be assessed whilst the patient is on treatment and for up to 6 weeks after completion of treatment in all patients. Patients completing 14 days of BKM120 treatment who either complete the follow up period or withdraw early fromor experiencing DLT will be evaluable for dose escalation decisions. Patients who withdraw early for reasons other than experiencing DLT (e.g. progression) will be considered non-evaluable for dose escalation and replaced as necessary to complete each cohort. Patients replaced for dose escalation earlier than the 14 days will be followed up and considered evaluable for toxicity analysis.

Expansion cohort and cohort 4
Secondary endpoints of changes in ^{18}F-Misonidazole and in blood flow will be based on ^{18}F-Miso PET-CT and perfusion CT scans prior to commencing BKM120 treatment and on the day that radiotherapy treatment commences. Patients who have data available from both scans will be considered evaluable for secondary endpoints. Patients withdrawn early or without scans will be considered non-evaluable for secondary endpoints and replaced as necessary to

complete each cohort. All patients who receive at least one dose of BKM120 will be followed up and will be considered evaluable for toxicity analysis.

7 SAMPLES FOR LABORATORY ANALYSIS

7.1 Samples to be analysed in local Trust's laboratories

Diagnostic Laboratories

Samples for haematology and biochemistry analysis will be labelled with standard patient identifiers and sent to the local hospital diagnostic laboratory. Results will be processed in the standard way and entered into the routine hospital reporting system. Samples will be stored, held reported and subsequently destroyed in accordance with standard local laboratory practice.

7.2 Samples to be sent to and analysed in a Central Laboratory

Research blood samples will be collected, processed and stored by the clinical study site in accordance with written instructions that will be provided in a separate sample handling manual. For phospho-Akt expression 20ml blood samples will be taken at the timepoints indicated in Sections 5.3 and 5.4. Samples will be retained and dispatched to a central laboratory for analysis.

The Chief Investigator or designee will apply for access to archival pathology material from the relevant Pathology Department. Any available specimens will be sent to a designated laboratory for central analysis. Separate shipping instructions will be provided.

7.3 Summary of research samples to be taken during the study

Assay/sample handling and storage will be managed according to separate written instructions in the Sample Handling Manual. Research assays will be performed according to separate laboratory SOPs.

7.4 Pharmacodynamic assays

The levels of phospho-Akt expression in normal tissues may reflect the efficacy of BKM120. Changes in phospho-Akt expression will be monitored during the trial using PBMCs taken at the timepoints indicated in Sections 5.3 and 5.4. Reductions in phospho-Akt expression of PBMC's following BKM120 treatment will be correlated with changes observed with the functional imaging investigations.

7.5 Molecular studies

Exploratory studies will aim to correlate response to BKM120 with activation of specific molecular pathways present in the archived, diagnostic, tumour biopsy sample. In cases where there is sufficient tumour tissue available, we will assess pAkt and PTEN levels and then the mutation status of RAS, PI3K and EGFR by PCR. In cases where there is insufficient tissue available to perform these investigations we will prioritise the assessment of pAkt levels.

7.6 Labelling and confidentiality of samples sent to central laboratories

All samples sent to analytical Laboratories will be labelled with the trial code, trial patient number, dosing cohort and date taken. Should a laboratory receive any samples carrying unique patient identifiers the recipient must immediately obliterate this information and re-label. The study site will be informed of their error.

7.7 Clinical reporting of research assay results

The results of the molecular assays are exploratory and are not intended to influence the individual patient's medical care. Findings will not be reported routinely to the responsible clinician except in the unlikely event that the result might be beneficial to the patient's clinical management.

7.8 Trial sample retention at end of study

The Chief Investigator has overall responsibility for custodianship of the trial samples. Laboratories are instructed to retain any surplus tumour samples pending instruction from the Chief Investigator on use, storage or destruction. It is possible that new or alternative assays may be of future scientific interest. At the end of the research study any surplus tumour samples may be retained for use in other projects that have received ethical approval. Hence, any surplus tumour samples may be transferred to a licensed tissue bank where they will be managed in accordance with

applicable host institution policies and the Human Tissue Act (HTA) requirements. Any excess blood samples will be destroyed once the study has been completed.

7.9 Withdrawal of consent for sample collection and/or retention

A patient may withdraw consent to take samples for research and/ or to retain and use their samples for research at any time without giving a reason. The Investigator must ensure that their wishes are recorded in the medical record and will inform the trials office accordingly. No further samples will be taken and any unused samples will be destroyed as per host institution policy.

8 INVESTIGATIONAL MEDICINAL PRODUCTS (IMPS)

Patients will be assigned to the appropriate BKM120 dose level according to the dose escalation scheme given below. BKM120 will not be dose escalated above 100mg od, as this dose has previously been established as the RPTD for future single agent studies (1). BKM120 will be supplied by Novartis and packaged, labelled and distributed by Fisher Clinical Services.

8.1 Dosing schedules: dose levels and BKM120 dose escalation plan

In this study, DLT is defined as the presence of the following:

1. Any ≥ grade 3 non-haematological toxicity (excluding nausea, vomiting or diarrhoea) that requires hospital admission or which does not resolve to ≤ grade 2 within 7 consecutive days of optimal treatment.
2. Any ≥ grade 3 nausea, vomiting or diarrhoea will be considered DLT only if any of them persist for >48 hours despite maximum supportive care.
3. ≥ Grade 3 pneumonitis
4. Any ≥ grade 4 haematological toxicity.
5. Mood deterioration from baseline DLT will be any grade ≥3 mood change if BL score of 2. DLT will be any grade ≥2 mood change if BL score of ≤ 1.

DLT will be considered to occur if the toxicity is attributed either to BKM120 or to its interaction with radiotherapy. Cohort 4 will only be opened if there is inadequate evidence that BKM120 alters tumour hypoxia or perfusion (see Section 12.1)

DL	RT	BKM120	BKM120 dose escalation plan
1	20Gy in 5 fractions (days 8 - 14)	50 mg od (days 1 to 14)	Three patients will be entered<br>If 0/3 have DLT proceed to DL2<br>If 1/3 have DLT expand dose level to a total of 6 patients. If none of the additional 3 patients develop DLT, proceed to DL2.<br>If any further patient(s) develop DLT (≥ 2/6) the MTD is exceeded and the trial will be terminated.
2	20Gy in 5 fractions (days 8 - 14)	80 mg od (days 1 to 14)	Three patients will be entered<br>If 0/3 have DLT proceed to DL3.<br>If 1/3 have DLT expand dose level to a total of 6 patients. If none of the additional 3 patients develop DLT, proceed to DL3.<br>If any further patient(s) develop DLT (≥ 2/6) the MTD will be defined as 50mg od.
3	20Gy in 5 fractions (days 8 - 14)	100 mg od (days 1 to 14)	Three patients will be entered<br>If 0/3 have DLT then the MTD is 100 mg od.<br>If 1/3 have DLT expand dose level to a total of 6 patients. If none of the additional 3 patients develop DLT, the RPTD is 100 mg bd.<br>If any further patient(s) develop DLT (≥ 2/6) the MTD will be defined as 80mg od.
Expanded cohort at RPTD	20Gy in 5 fractions (days 8 - 14)	MTD dose (days 1 to 14)	Once the MTD has been reached a further 6 patients will be treated at the MTD of BKM120 with palliative RT.
4	20 Gy in 5 fractions (days 22 – 28)	At MTD (days 1 to 28)	This cohort will only be used if there is no evidence of BKM120 induced changes in the tumour microenvironment on functional imaging in cohorts 1-3. A total of six patients will be entered into this cohort.

8.2 Management of drug administration

BKM120 will be supplied as 10mg and 50mg hard gelatin capsules. BKM120 will be administered on a continuous daily dosing from the morning of day 1, until the morning of the final RT treatment. A flat scale of mg/day will be used and the dose will not be adjusted to body weight or surface area. The dose levels and total number of days of BKM120 treatment for cohorts 1-3 and for cohort 4 are given in section 8.1.

The potential CYP3A4 interaction associated with BKM120 means that patients must avoid consumption of Seville orange (and juice), grapefruit (and juice), and exotic citrus fruits from 7 days prior to the first dose of study drug and during the entire study period. Regular orange juice is allowed.

The following general guidelines should be followed for BKM120 administration:

- Patients should be instructed to take the dose of BKM120 once daily in the morning at approximately the same time each day
- BKM120 should be taken one hour following a light breakfast
- Patients should not eat for 2 hours after the administration of each BKM120 dose
- BKM120 should be taken with a glass of water. Patients should swallow the capsules as a whole and not chew them
- Patients taking medicinal products that may alter the pH of the upper GI tract should follow guidance in section 9.3

If vomiting occurs during the course of treatment, re-dosing is not permitted. Missed doses will not be made up. The next dose must be taken as scheduled.

8.3 Special precautions

Non-infectious pneumonitis

Lung changes compatible with pneumonitis have not been observed in the preclinical setting.
Based on the literature, this class of PI3K inhibitors has not previously been associated with the development of pneumonitis.

In ongoing clinical trials with BKM120 in the single agent setting, two cases of pneumonitis have been reported. In study BKM120X2101 one patient experienced Pneumonitis grade 2 eight weeks after the first dose of BKM120 at 100mg which resolved in 7 days after antibiotic therapy and discontinuation of the study treatment due to fatigue. In the Japanese study BKM120X1101 one case of pneumonitis has been reported in one patient after one month of treatment with 100 mg of BKM120. The patient experienced pneumonitis grade 5 which was concomitant with progression of the underlying malignancy including the progression of existing lesions as well as the appearance of new lesions in combination with increasing pleural effusion (please see current IB for more details). The data currently available does not enable a clear assessment about the causal relationship of pneumonitis with BKM120 treatment.

Pneumonitis is a common side effect associated with thoracic radiotherapy treatment. The palliative dose of radiation used in this study may be associated with grade 1 or 2 pneumonitis but would not be expected to cause more severe pneumonitis. The development of grade 3 pneumonitis would be unusual, and will be considered to reflect a dose limiting toxicity of BKM120 resulting in the patient discontinuing BKM120 treatment.

Patients will be asked about new respiratory symptoms at each visit. Patients with suspected pneumonitis will be investigated as deemed appropriate by the investigator. This may involve chest CT scans and lung function tests as well as referral to a respiratory physician. Symptomatic management with steroids and oxygen will be initiated as required.

Mood alteration

There is some pre-clinical evidence that modulation of the AKT/GSK3 signalling pathway by neurotransmitters is important for the regulation of behaviour. Preclinical studies conducted in rats to investigate the effect of BKM120 of different neurotransmitters have shown that repeated administration of BKM120 resulted in an enhanced decrease in glutamate, dopamine, serotonin, and epinephrine as well as in an enhanced increase in GABA and HIAA.

As of September 2012, approximately 4% of patients treated with BKM120 at 100mg od developed G3/4 mood alteration. In order to lower the risk of BKM120 induced mood alteration, patients with a past or current history of significant psychiatric disorders will be excluded from the study. Patients will also have to satisfactorily complete two different mood questionnaires (PHQ-9 and GAD-7) in order to be eligible for entry. Patient’s mood will be carefully monitored throughout the study using these questionnaires at each visit.

The following grading system will be used for this study:

PHQ-9			GAD-7		
Score	Severity	CTCAE grading	Score	Severity	CTCAE grading
0-4	None	Normal	0-4	None	Normal
5-9	Mild	Grade 1	5-9	Mild	Grade 1
10-19	Moderate	Grade 2	10-14	Moderate	Grade 2
20-27	Severe	Grade 3	≥15	Severe	Grade 3
* Note: The grading guidance above may be overruled at the discretion of the Chief Investigator following a psychiatric review.					

At Screening, a patient as judged by the investigator or a psychiatrist to be ineligible or who meets the cut-off score of ≥ 12 in the PHQ-9 or a cut-off of ≥ 15 in the GAD-7 mood scale, respectively, or selects a positive response of ‘1, 2, or 3’ to question number 9 regarding potential for suicidal thoughts ideation in the PHQ-9 (independent of the total score of the PHQ-9) or who otherwise exhibit any suicidal ideation regardless of the question 9 scoring will be excluded from the study.

During the study, patients who develop mood alteration that constitutes a DLT, or who indicate a positive response by selecting '1, 2, or 3' to question number 9 on the PHQ-9 or who otherwise exhibit any suicidal ideation regardless of the question 9 scoring must see a psychiatrist for diagnosis and determination of most appropriate medical treatment. Patients will then be followed twice weekly by patient self-rating mood scale and will be seen weekly by the psychiatrist until the symptoms have resolved to ≤ grade 1 or baseline.

Hyperglycaemia

The PI3K/Akt pathway plays a significant role in regulating glucose metabolism, particularly by regulating glucose transport into adipocytes and muscle tissue. Hyperglycaemia is therefore considered an 'on target' effect of BKM120. Transient increases in plasma glucose levels have been commonly reported in patients taking BKM120.

Patients with poorly controlled diabetes (HbA_{1c} > 8 %) or a fasting plasma glucose (FPG) ≤ 120 mg/dL or ≤ 6.7 mmol/L at baseline will be ineligible for trial entry. FPG will be assessed at each subsequent visit. Hyperglycaemia will be managed with appropriate diabetic medication in consultation with endocrinology advice. Since *in vitro* studies have shown that BKM120 may inhibit insulin-stimulated glucose uptake, treatment is likely to involve the use of oral anti-diabetic medication such as metformin which increases muscle and fat glucose uptake. BKM120 will be continued unless grade 3 hyperglycaemia develops (FPG > 250 - 500 mg/dL) [> 13.9 - 27.8 mmol/L]) at which point it will be discontinued. If grade 3 hyperglycaemia lasts for longer than 7 days despite optimal treatment it will be considered a DLT.

Cardiac

Cardiac safety studies, conducted in vitro and in vivo, indicate a minimal risk of an electrophysiological effect of BKM120. As of September 2012 adverse cardiac events following BKM120 treatment were rare. Nevertheless, cardiac monitoring will be undertaken with an ECG prior to commencing BKM120 and repeated during the trial. Concomitant use of any medications known to cause QT prolongation is prohibited (a list of such medications is provided in Appendix 3).

Liver

Liver function test (LFT) alterations have been observed in patients taking BKM120. These have mostly involved temporary increases in transaminase enzymes (ALT and/or AST). Transaminase elevations typically occur during the first 6 to 8 weeks of treatment start. Only a few of the patients with LFT alterations had other simultaneous observations like bilirubin increase or clinical symptoms related to impaired hepatic function. A recent safety review across Novartis-sponsored trials has identified several potentially drug-induced liver toxicity (DILI) cases in the absence of cholestasis and other explanatory causes. This protocol has applied a conservative selection criteria to restrict the inclusion of patients with abnormal LFTs. Liver function will be monitored throughout the study and BKM120 discontinued if bilirubin >2.0 x ULN or AST/ALT >3.0 x ULN occurs and hepatology advice will be sought and appropriate follow-up assessments will be carried out to event resolution or stabilization (for patients with Gilbert Syndrome these dose modifications apply to changes in direct bilirubin only).

Follow-up assessments may include (as clinically indicated):

- Evaluate if associated with the appearance or worsening of clinical symptoms of hepatitis or hypersensitivity such as fatigue, nausea, vomiting, right upper quadrant pain or tenderness, fever, rash or eosinophilia, or other organ involvement.
- Obtain bilirubin, serum Alkaline Phosphatase (ALP), creatine phosphokinase (CPK), lactate dehydrogenase (LDH), and blood count with differential to assess eosinophilia.
- Perform liver imaging (ultrasound, MRI, or CT) to evaluate liver disease including metastasis or new lesions, obstruction/compression, etc.
- Perform viral hepatitis and other serology tests:
  - Hepatitis C (HCV) serology and viral RNA, Hepatitis B (HBV) serology and viral DNA, Hepatitis A (HAV) Immunoglobulin M (IgM) and HAV total
  - Hepatitis E (HEV) serology: IgM and IgG, viral RNA
  - Herpes Simplex Virus (HSV), Cytomegalovirus (CMV), Epstein-Barr viral (EBV) serology
- Verify and record the use of concomitant medications, acetaminophen, herbal remedies, and other over the counter medications, or putative hepatotoxins, on the concomitant medications report form.

- Liver biopsy as clinically indicated to assess pathological change and degree of potential liver injury
- LFTs should be followed-up weekly until resolve to ≤ grade 1, baseline or stabilisation (no CTCAE grade change over 4 weeks) and outcome documented on the respective AE and lab chemistry pages.

This guidance has been updated since the completion of the escalation phase. During the escalation phase grade 3 bilirubinaemia or transaminitis events would result in the discontinuation of BKM120 and if the event lasted for longer than 7 days qualify as a DLT.

8.4 Dose modification

If a patient is unable to tolerate BKM120 due to side-effects, then BKM120 will be stopped. Scheduled visits and all assessments should continue to occur except the dosing of BKM120. The BKM120 dose will not be reduced. For replacement of non-evaluable patients see Section 6.1.

8.5 Compliance

Patients will be asked to bring all their unused/remaining supplies of BKM120 to specified clinic visits for checking by the research team.

8.6 Management of overdose

There is no specific antidote for BKM120. Patients experiencing toxicities upon miss-dosing or overdosing will be treated at the discretion of the investigator with adequate supportive care and followed until recovery.

8.7 Dose escalation review

The Trial Management Group will decide upon dose escalation 6 weeks after the final patient in each cohort has completed radiotherapy treatment. All decisions will be documented. Emerging safety data will be reviewed throughout the trial and appropriate measures taken to ensure the safety of participants.

9 OTHER TREATMENTS

9.1 Background systemic therapy

There will be no background therapy.

9.2 Support medication

The patients recruited to this study will have advanced NSCLC. It is therefore imperative that appropriate palliative care treatments are made available to them as per local practice. Additional side-effects associated with either BKM120 or radiotherapy treatment will be treated as per local practice.

BKM120 induced diarrhoea should be considered once other potential causes such as food, comorbidity, or concomitant medication have been considered and treated. Patients will be monitored for signs of dehydration and instructed to take preventative measures against dehydration. Concomitant medication to treat diarrhoea such as loperamide given at a standard dose will be considered for Grade 1-2 diarrhoea along with oral hydration and dietetic measures. More severe diarrhoea should be treated appropriately at the investigators discretion and may include IV fluid administration.

Patients will be monitored for skin reactions at each planned visit. Although preclinical experiments with BKM120 have demonstrated that BKM120 has no potential phototoxic effect, a small number of patients have experienced some phototoxicity. Patients will therefore be advised to avoid sun exposure during treatment with BKM120, especially when they have already experienced rash or other skin toxicities. Skin rash will be managed at the investigators discretion and may include the use of anti-histamines and corticosteroids.

Examples of additional supportive care procedures are outlined below.

Nausea and vomiting	Anti-emetic therapy is appropriate to manage treatment related nausea and should be given as per local practice.

Pneumonitis	Therapy with steroids and oxygen is appropriate to manage treatment-related pneumonitis and may be given as per local practice.
Oesophagitis	Therapy with antacids, proton pump inhibitors and analgesics is appropriate to manage treatment-related oesophagitis and may be given as per local practice.<br><br>Patients may require IV hydration if they suffer protracted oesophagitis resulting in significant dysphagia and odynophagia.
Rash	BKM120 induced rash may be managed with anti-histamine and steroid treatment as required

9.3 Concomitant medication and non-drug therapies

Concomitant therapies may be given as medically indicated. Details (including indication, doses, frequency and start / stop dates) of concomitant medication will be documented in the medical record at entry to the trial and at each follow-up visit.

BKM120 is characterised by a pH-dependent solubility. Medicinal products that alter the pH of the upper Gastro-Intestinal (GI) tract may alter the solubility of BKM120 and hence its bioavailability. These agents include, but are not limited to, proton-pump inhibitors (e.g. omeprazole), H2 antagonists (e.g. ranitidine) and antacids. BKM120 should be dosed in a staggered manner at least 1 hour before or 10 hours after dosing with medicinal products that may alter the pH of the upper GI tract.

Prolonged corticosteroid treatment

Prolonged corticosteroid treatment equivalent to dexamethasone 4mg od does not significantly alter the pharmacokinetics of BKM120. However, these treatments should be used with caution in order to avoid inducing hyperglycaemia.

9.4 Prohibited therapies

Patients must not be prescribed any other anti-cancer or investigational therapies, other than the study treatments, whilst participating in this study.

CYP450 metabolised drugs

BKM120 is a weak, reversible inhibitor of CYP3A4/5, CYP2C8, CYP2C9 and 2C19. With the data available, it is not possible to confirm whether such interactions will occur in patients. Therefore, caution will be used in the administration of concomitant medications known to be metabolized by CYP3A4/5, CYP2C8, CYP2C9 and CYP2C19. Patients receiving such medications must be monitored for potentiation of toxicity due to any individual concomitant medications, and may require dose titration or reduction of the drug substrate. A list of CYP3A4 and CYP2C substrates to be used with caution is given in Appendix 2. Particularly, caution should be used when BKM120 is co-administered with drugs that are sensitive substrates and/or have a narrow therapeutic index.

Patients taking a statin may continue this medication during the trial if it is considered desirable for them to remain on an anti-hypercholesterolaemic drug but should be monitored carefully.

QT interval prolonging drugs

Drugs which may prolong the QT interval and which are prohibited, or which should only be used with caution are shown in Appendices 3 and 4 respectively.

Moderate and strong CYP3A inducers and inhibitors

In vitro metabolism studies suggest that oxidative metabolism of BKM120 is predominantly mediated by CYP3A4, with only minor contributions of CYP1A2 and UGT1A4. Coadministration of BKM120 with strong and moderate CYP3A4

inhibitors is predicted to increase the systemic exposure to BKM120 by more than 5-fold and more than 2-fold, respectively.
Based on in vitro studies, co-administration of BKM120 with CYP3A4 **inducers** such as enzyme-inducing anti-epileptic medication, is predicted to decrease the systemic exposure to BKM120, thereby increasing the risk of exposing the patient to subtherapeutic drug levels.

A list of prohibited drugs is given in Appendix 5.

Herbal Medications
Herbal preparations/medications are not allowed throughout the study. These herbal medications include, but are not limited to: St. John's wort, Kava, ephedra (ma huang), gingko biloba, dehydroepiandrosterone (DHEA), yohimbe, saw palmetto, ginseng. Patients should stop using these herbal medications 7 days prior to first dose of study drug.

Warfarin and coumarin derivatives
Therapeutic doses of warfarin sodium (Coumadin®) or any other coumarin-derivative anticoagulants are not permitted. Treatment with heparin, low molecular weight heparin, or fondaparinux is allowed.

10 RADIOTHERAPY

Patients will be treated with palliative radiotherapy using 20Gy in 5 fractions. This dose and fractionation scheme reflects current local practice.

Radiotherapy planning, delivery and treatment verification will be carried out as per local policy. Participation in the trial will not involve any additional imaging relating to treatment planning or delivery.

10.1 Radiotherapy simulation and planning

Patients will be positioned on an immobilisation couch in the supine position and a planning CT scan performed as per departmental protocol. Treatment will be planned using 6 to 15 MV photons. The photon energy which results in the optimal RT dose distribution within the target volume and which minimises dose to non-target tissues will be selected.

The radiotherapy treatment volume will be planned as per local protocol. The treatment will be planned so that the disease considered to be responsible for the patients' symptoms will be encompassed. An adequate margin (typically 2cm) will be added to allow for set-up variations and tumour movement.

10.2 Radiotherapy field arrangement and prescription

The radiotherapy treatment fields will usually be arranged as a parallel opposed pair. Multileaf collimators will be used to shield normal tissue as required. The maximum field area will not exceed $200cm^2$.

Radiotherapy doses of 20Gy in 5 fractions will be prescribed for all patients in the study. The radiotherapy will be prescribed according to ICRU guidance and local practice.

10.3 Radiotherapy treatment delivery

To ensure that the radiotherapy treatment is targeted accurately, verification imaging will be performed in accordance with local practice. Radiotherapy quality assurance will be as per local policy.

10.4 Radiotherapy dose constraints

The radiotherapy doses used within this study are within the tolerance doses of all critical normal tissues such as the lung and spinal cord. Therefore no specific dose constraints will be applied for the purposes of radiotherapy planning.

11 DRUG MANAGEMENT

11.1 Drug supplies

BKM120 will be supplied by Novartis as 10mg and 50mg hard gelatine capsules, packaged as 35 capsules per bottle in unlabelled HDPE bottles. The bottles will be appropriately labelled in accordance with all applicable regulatory requirements, by Fisher Clinical Services.

11.2 Drug ordering

Initial supplies of BKM120 are sent out by Fisher Clinical Services after they have been informed by OCTO that all approvals are in place. Subsequent supplies will be ordered by OCTO. Pharmacy should request additional shipments by contacting the BKM120 trial office.

11.3 IMP Receipt

Study drug must be received by a designated person at the study site, handled and stored safely and properly, and kept in a secured location to which only the investigator and designated assistants have access.

11.4 Handling and storage

Upon receipt, BKM120 should be stored according to the instructions specified on the drug labels. It will be stored & handled in pharmacy in accordance with local procedures.

11.5 Labelling

BKM120 will be supplied, appropriately labelled in accordance with all applicable regulatory requirements, by Fisher Clinical Services to the study site.

11.6 Dosing dispensing

BKM120 will be dispensed as 35 x 10mg and 35 x 50mg hard gelatin capsules in HDPE bottles In accordance with local procedures for dispensing IMPs. For all cohorts sufficient drug will be dispensed at day -1 for the duration of the trial. See table below:

Cohort	No. 10mg Bottles/pt	No. 50mg Bottles/pt
1 (50mg)	0	1
2 (80mg)	2	1
3 (100mg)	0	1
Expanded dose (50mg)	0	1
Expanded dose (80mg)	2	1
Expanded dose (100mg)	0	1
4 (50mg)	0	1
4 (80mg)	3	1
4 (100mg)	0	2

11.7 Drug accountability

Full drug accountability records must be maintained for BKM120 using the Drug Dispensing Log provided. The hospital may amend the Drug Dispensing Log provided or use their own documentation if it captures all the information requested on the Drug Dispensing Log. This must be approved by the trials office prior to use.

The Drug Dispensing Logs must be available for inspection by the monitor at every monitoring visit. At the conclusion of the study the overall numbers of drug shipped to the centre, the number dispensed and the number destroyed or returned will be provided by the pharmacy. An account must be given of any discrepancy.

11.8 Drug returns from patients

Patient returns of BKM120 should be returned to pharmacy for counting and recording in the patient's Dispensing Log. Returns may then be destroyed on site according to local practice (verification by monitor not required).

11.9 Drug destruction

At the end of the study, once authorised to do so, any unused drug should be disposed of at site according to local hospital policy. A dated certificate of disposal should be completed. The original should be placed in the Pharmacy File and a copy emailed/faxed to the Trials Unit. Any patient returns should be disposed of at site according to local hospital policy

11.10 Occupational safety

The product is not expected to pose an occupational safety risk to site staff under normal conditions of use and administration.

12 EVALUATION OF RESPONSE TO BKM120

Imaging investigations will be aimed at identifying changes in tumour physiology following BKM120 treatment rather than assessing morphological changes in the tumour following radiotherapy treatment. Patients' will undergo ^{18}F-Miso PET-CT and perfusion CT scans as per departmental protocol. The ^{18}F-Miso PET-CT imaging procedure will involve three scans to be performed after injection and then at approximately 2 and 4 hours after injection. During this time, up to 4 blood samples will be obtained from a venous cannula. Less than 5ml of blood is generally required for each test and it is unlikely that more than 15ml of blood will be required in total.

12.1 Tumour assessment

Response to BKM120 treatment will be based upon changes in tumour hypoxia and perfusion as detected by ^{18}F-Miso PET-CT scans and perfusion CT scans respectively. These imaging investigations will be performed the day prior to commencing BKM120 treatment, and on the day that radiotherapy treatment commences. The imaging investigations will be repeated prior to the patient receiving their first fraction of radiotherapy.

The rationale for this timing is to observe whether BKM120 can reduce tumour hypoxia and increase tumour perfusion, since both of these changes would be likely to increase the efficacy of radiotherapy treatment.

Establishing the appropriate method for analysis of both imaging techniques is part of the research question. Once established the same method of assessment and the same technique will be used throughout the trial. Changes in tumour-to-blood ratio (TBR) mean and volume will be identified with ^{18}F-Miso PET-CT imaging and changes in tumour blood flow, blood volume, and mean transit time assessed with perfusion CT imaging.

If there is no change in tumour hypoxia or perfusion following one week of BKM120 treatment, patients will be recruited to cohort 4 where they will receive three weeks of BKM120 at the MTD prior to commencing radiotherapy.

Perfusion CT and dynamic ^{18}F-Misonidazole PET-CT scans will be performed as summarised in Sections 5.3 and 5.4. Each patient will be classified as a 'responder' or 'non-responder' on the basis or their pre- and post- BKM120 imaging. Those patients whose imaging demonstrates the presence of a physiological response on either perfusion CT and/or ^{18}F-Misonidazole PET-CT will be classified as a responder whilst those that do not show a response on either imaging modality will be classed as a non-responder.

Patients will only be recruited to cohort 4 if more than 50% of the patients treated with the MTD of BKM120 (n = 9 to 12 evaluable) are defined as non-responders. The following is considered guidance and the statistical analysis of the imaging endpoints will be detailed in the SAP with approval from the DSMC/TSC.

Perfusion will be assessed on the basis of perfusion CT. Perfusion parameters including blood flow (BF), blood volume (BV) and mean transit time (MTT) will be measured using standard commercially supplied software. Patients will be classified as 'responders' if the BF is increased and/or MTT is reduced from the baseline measurements by more than 25%. This is in keeping with published data on the use of BF and MTT to predict response to chemoradiotherapy (10). . Recent information from research of F-MISO in head and neck cancer patients (11) has reported a mean (SD) tumour to muscle (TMR) volume of 3% (14%). Extrapolating from this data and following discussion with the DSMC/TSC, patients will be classified as "responders" using the minimum detectable change (MDC) (12) method if their differences are ≥ 10% for at least one of the hypoxia parameters TBR mean or TBR volume.

12.2 Tumour response

Imaging investigations performed in this study will aim to identify changes in tumour physiology rather than tumour measurements. Tumour responses will therefore not be expected or formally recorded.

12.3 Other definitions of outcome:

Toxic death: Any death to which drug toxicity is thought to have a major contribution.
Early death: Death during the first three weeks of treatment that is not a toxic death.

13 ASSESSMENT OF SAFETY

The Investigator will monitor each patient for clinical and laboratory evidence of adverse events on a routine basis throughout the study. Should an Investigator become aware of any study drug related SAEs following this period these must also be reported as stated below. Adverse event monitoring starts from the time the patient receives any of the research procedures until they complete the trial. All reportable AEs will be followed to a satisfactory conclusion. Any reportable AEs that are unresolved at the patient's last visit in the study are followed up by the Investigator for as long as medically indicated, but without further recording in the CRF.

All AEs reported to the trial office will be processed by the trial office according to internal OCTO standard operating procedures. The trial office may request additional information for any AE as judged necessary.

13.1 Adverse Event Definitions

An Adverse Event or experience (AE) is any untoward medical occurrence in a patient or research study subject temporally associated with the administration of an investigational medicinal product (IMP) or a comparator product, whether or not considered related to the IMP or a comparator product. An AE can therefore be any unfavourable and unintended sign, symptom, disease (new or exacerbated) and /or significant abnormal laboratory or physiological observation temporally associated with the use of a medicinal product.

A Serious Adverse Event (SAE) is any AE, regardless of dose, causality or expectedness, that:

• **Results in death**	
• **Is life-threatening**	This refers to an event in which the subject was at risk of death at the time of the event. It does not refer to an event, which hypothetically might have caused death, if it were more severe.
• **Requires in-patient hospitalisation or prolongs existing inpatient hospitalisation**	In general, hospitalisation signifies that the subject has been detained (usually involving at least an overnight stay) at the hospital or emergency ward for observation and/or treatment that would not have been appropriate in the physician's office or out-patient setting. Complications that occur during hospitalisation are AEs. If a complication prolongs hospitalisation or fulfils any other serious criteria, the event is serious. When in doubt as to whether hospitalisation occurred or was necessary, the AE should be considered serious.
• **Results in persistent or significant incapacity or disability**	This means a substantial disruption of a person's ability to conduct normal life functions. It does include experiences of relatively minor medical significance or accidental trauma (e.g. sprained ankle), which do not constitute a substantial disruption.
• **Is a congenital anomaly or birth defect**	
• **Is any other medically important event**	Defined as an event that may jeopardise the patient or may require intervention to prevent one of the outcomes listed above. Any new primary cancer must be reported as an SAE.

An Adverse Drug Reaction (ADR) is an AE which is considered to be causally related to any dose of the IMP. This means that a causal relationship between the IMP and the AE is at least a reasonable possibility, i.e., the relationship cannot be ruled out.

An Unexpected Drug Reaction is an adverse drug reaction, the nature or severity of which, is not consistent with applicable product information.

A Suspected Unexpected Serious Adverse Drug Reaction (SUSAR) is a serious adverse reaction, the nature or severity of which is not consistent with the applicable product information (e.g. Investigator's Brochure for an unapproved investigational product or SPC for an approved product).

13.2 Clinical laboratory abnormalities and other abnormal assessments as AEs and SAEs

Abnormal laboratory findings (e.g., clinical chemistry, haematology, urinalysis) or other abnormal assessments (e.g., ECGs, X-rays and scans) that are judged by the investigator as clinically significant will be recorded as AEs or SAEs if they meet the definitions given above. By definition, **all clinically significant Grade 3 or 4 laboratory abnormalities should be reported as SAEs.**

Clinically significant abnormal laboratory findings or other abnormal assessments that are detected during the study or are present at baseline and significantly worsen following the start of the study will be reported as AEs or SAEs. However, clinically significant abnormal laboratory findings or other abnormal assessments that are associated with the disease being studied, unless judged by the investigator as more severe than expected for the patient's condition, or that are present or detected at the start of the study and do not worsen, will not be reported as AEs or SAEs.

The investigator will exercise his or her medical and scientific judgment in deciding whether an abnormal laboratory finding or other abnormal assessment is clinically significant.

13.3 Determining adverse event causality

The Investigator will assess and classify the relationship of an AE to the trial IMP as follows:

Classification	Relationship	Definition
**drug-related**	**Almost certainly**	• Starts within a time related to the study drug administration *and*<br>• No obvious alternative medical explanation.
	**Probably**	• Starts within a time related to the study drug administration *and*<br>• Cannot be reasonably explained by known characteristics of the patient's clinical state.
	**Possibly**	• Starts within a time related to the study drug administration *and*<br>• A causal relationship between the study drug and the adverse event is at least a reasonable possibility.
**not drug related**	**Unlikely**	• The time association or the patient's clinical state is such that the study drug is not likely to have had an association with the observed effect.
	**Unrelated**	• The AE is definitely not associated with the study drug administered.

The Investigator must endeavour to obtain sufficient information to confirm the causality of the adverse event (i.e. relation to surgery, study drug, background treatment, other illness, progressive malignancy etc) and give their opinion of the causal relationship between each AE and study drug. This may require instituting supplementary investigations of significant AEs based on their clinical judgement of the likely causative factors and/or include seeking a further specialist opinion.

13.4 Expected adverse events

Section 5 of the IB for BKM120 lists all the expected side effects associated with the use of BKM120. A copy of this document must be held in the Site File for reference.

Thoracic radiotherapy treatment is often associated with side effects such as fatigue, oesophagitis, pneumonitis, and nausea. However with the palliative doses of radiotherapy that will be used in this trial, these side effects are usually mild and resolve within a few weeks of completing treatment. If a patient experiences more severe side effects, the Investigator will decide whether these have occurred as a result of BKM120 or its interactions with radiotherapy treatment, based on the severity of symptoms and other clinical information such as the radiotherapy volumes.

13.5 Suspected Unexpected Serious Adverse Drug Reactions (SUSARs)

A Suspected Unexpected Serious Adverse Drug Reaction (SUSAR) is a serious adverse reaction, the nature or severity of which is not consistent with the applicable product information (e.g. Investigator's Brochure for an unapproved investigational product or SPC for an approved product). SUSARS therefore are suspected to be at least possibly related to the study agent, fulfil the definition of a SAE, the nature and severity of which are not consistent with the applicable product information. In addition, other safety issues qualify for expedited reporting where they might materially alter the current risk assessment of an IMP or be sufficient to change IMP administration or the overall conduct of the trial. Responsibility for expedited safety reporting to responsible authorities will be done by the trials office using their own internal OCTO SOPs.

13.6 Expedited reporting of SAEs

The following SAE reporting requirements apply regardless of the Investigator's assessment of the causality or expectedness of the SAE. All SAEs should be reported on the trial SAE paper report form (see SAE report form and completion guidelines).

SAE reports must be sent within 24 hours of site staff becoming aware of the event to either:

PV Email: **octo-safety@oncology.ox.ac.uk**
OCTO Fax: **01865 617010**

- If the SAE has not been reported within the specified timeframe, a reason for lateness must be provided when sending the SAE Report Form.

- Investigators should also adhere to their local Trust policy for incident and SAE reporting in research.

-OCTO will be responsible for reporting all SAEs to Novartis within 24 hours of receiving an SAE report.

13.7 Follow-up of Serious Adverse Events

If new or amended information on a reported SAE becomes available, the Investigator should report this on a new SAE Report Form using the completion guidelines. Extra annotated information and/or copies of test results may be provided separately. If using the original form to notify further information, all new or amended information must be initialled and dated so that all changes are clearly identified.

Follow up will continue until all the necessary safety data for the event has been gathered. Follow up information must be sent to the trials office within one working day of the site becoming aware. Any SAE that is ongoing when a subject completes his/her participation in the trial must be followed until any of the following occurs:

- The event resolves or stabilizes;
- The event returns to baseline condition or value (if a baseline value is available);
- The event is attributed to other agent(s) or to factors unrelated to study conduct.

SAEs that are considered to be definitely unrelated to the trial intervention will not be followed up and monitored. SAEs must be reported until 6 weeks after end of treatment.

13.8 Reporting Adverse Events on the CRF

All AEs, including Serious AEs (except those described in section 13.9) must be recorded on the case report forms (CRF) for that patient. Please note that AEs are recorded on eCRFs within OpenClinica and SAEs are additionally recorded on the paper SAE report form and emailed/faxed to OCTO. The information provided will include date of onset, event diagnosis (if known) or sign/symptom, severity, time course, duration and outcome and relationship of the AE to study drug. Any concomitant medications or other any therapy used to treat the event must be listed. The Investigator will provide an "other" cause for serious AEs considered to be unrelated to the study drug. Sites should ensure data entered into the CRF is consistent with the SAE report information where applicable.

Each separate AE episode must be recorded. For example, if an AE resolves completely or resolves to baseline and then recurs or worsens again, this must be recorded as a separate AE. For AEs to be considered intermittent, the events must be of similar nature and severity

The NCI CTCAE Version 4.0 must be used to grade each AE, and the worst grade recorded.

13.9 Events exempt from being reported as AE/ SAEs

This section specifies adverse events that do not require reporting providing the Investigator is certain that they are as expected given the natural course of the disease under study and /or expected outcomes of any background routine standard of care. The event must be reported if the Investigator cannot exclude the possibility that any trial intervention (including tests and other procedures) might be causally implicated or if the frequency, severity or pattern of events is not as expected for the patient's condition.

Progression of underlying disease

Disease progression and resultant death will be captured on the eCRF. Adverse events including hospitalisation and/or death that are clearly consistent with disease progression will not be reported as individual AE/SAEs. Clinical symptoms of progression will only be reported as adverse events if the symptom cannot be determined as exclusively due to the progression of the underlying malignancy, or does not fit the expected pattern of progression.

Every effort should be made to document the objective progression of underlying malignancy. In some cases, the determination of clinical progression may be based on symptomatic deterioration. For example, progression may be evident from clinical symptoms, but is not supported by tumour measurements. Or, the disease progression is so evident that the investigator may elect not to perform further disease assessments.

Death on study

The patients enrolled into this study will have incurable NSCLC which is associated with a poor prognosis. It is therefore likely that some patients will die as a result of their underlying malignancy whilst enrolled in this study. Death due to NSCLC will be recorded on the Death eCRF form providing no causal relationship with BKM120 is suspected. The investigator will state whether the death was expected or unexpected.

13.10 Informing Investigators of new safety information

The Chief Investigator will ensure that all investigators are kept informed in a timely manner, as new safety profile information becomes available. Investigators are responsible for briefing their study team and onward transmission to their local ethics committees and/or R&D office as appropriate.

14 PREGNANCY

Pregnancies (in a participant or partner) occurring whilst taking BKM120 treatment must be reported. A pregnancy notification form should be completed and emailed/faxed to the trial office within the same timelines as an SAE. All reported pregnancies should be followed and the outcome reported using the same form.

Note: Pregnancy is only considered an AE/SAE if there is reason to believe it may be the result of an interaction between the study IMP and the contraceptive used.

Women who become pregnant must be withdrawn from BKM120 treatment at the earliest opportunity.

All reported pregnancies should be followed up until term. If the outcome of the pregnancy meets any of the criteria for seriousness, it must also be reported as an SAE. Examples of pregnancy outcomes that are SAEs include reports of:

- congenital anomalies or developmental delay, in the foetus or the child.
- foetal death and spontaneous abortion
- suspected adverse reactions in the neonate that are classified as serious

15 DEFINING THE END OF TRIAL

For this study the end of the trial is defined as the last visit of the last patient enrolled in the trial i.e. approximately 6 weeks post completion of BKM120 plus radiotherapy.

The sponsor and the Chief Investigator reserve the right to terminate the study earlier at any time. In terminating the study, they must ensure that adequate consideration is given to the protection of the participants' best interests.

16 STATISTICAL CONSIDERATIONS

16.1 Sample size determination

If a MTD is reached, it is anticipated that between 6 and 18 evaluable patients will be required for the dose escalation phase of the study. Once the MTD has been reached an expanded cohort of 6 additional patients will be treated at the MTD of BKM120 with palliative RT. Furthermore, an optional cohort of 6 patients will receive 3 weeks of BKM120 prior to RT, bringing the total patient number for the study from 18 to 30.

<u>Dose escalation phase</u>
Between 2 and 18 evaluable patients will be recruited to the dose escalation phase. The aim of this Phase I study is to define the MTD of BKM120 when used in combination with palliative thoracic radiotherapy. The starting dose will be 50mg od. Unless DLT occurs this will be escalated to 80mg od and then 100mg od. There will be no further dose escalation beyond 100mg od. The study uses a standard 3+3 cohort design. Three patients will be entered at each dose level. At minimum, the criteria for dose escalation will be that if, by 6 weeks after the 3rd patient in each cohort has completed treatment there is no DLT, then the study will proceed to the next dose level. There will be a continuous evaluation of safety data, and dose escalation will be reviewed in response to any emerging safety information, with the Chief Investigator reserving the right to prolong the duration between cohorts as appropriate. The first cohort of patients will be given the starting dose of 50mg od, consideration will be given to stopping the recruitment early whether 2 or 3 DLTs are observed – consequently the minimum number of patients recruited is 2. If no DLT is observed, 3 patients will be entered at the next higher dose level. If 1 DLT is observed, an additional 3 patients will be entered at the same dose level. This process will be repeated until MTD is reached. This is the dose level at which no more than 1 of 6 evaluable patients or 0 of 3 evaluable patients experience a DLT. As a result, the maximum number of evaluable patients that could be recruited is 18 (6 evaluable patients by 3 dose levels)

<u>Dose expansion cohort</u>
To investigate the effect of BKM120 on tumour hypoxia and perfusion, an additional expansion cohort of a further six patients will be treated with BKM120 in combination with radiotherapy at whichever cohort (1-3) corresponds to the MTD.

<u>Dose cohort 4</u>
Six patients will be recruited to cohort 4 and will receive 3 weeks of BKM120 at MTD dose prior to RT if cohorts 1-3 have been unable to demonstrate that BKM120 causes changes in tumour hypoxia and perfusion as described in Section 12.1.

The study is planned to include a total of 2-30 patients. All patients will be scheduled to receive the same prescribed dose of radiotherapy.

16.2 Definition of primary and secondary endpoints

<u>Dose escalation phase</u>

The maximum tolerated dose (MTD) is defined as the highest dose of BKM120 in combination with radiotherapy at which no more than 1 of 6 evaluable patients or 0 of 3 evaluable patients experiences a DLT.

Expansion cohort and cohort 4
Changes in ^{18}F-Misonidazole retention and in blood flow between pre and post BKM120 treatment are defined as response to BKM120 treatment based upon changes in tumour hypoxia and perfusion as detected by ^{18}F-Miso PET-CT scans and perfusion CT scans respectively. Each patient will be classified as a 'responder' or 'non-responder' on the basis or their pre- and post- BKM120 imaging. Those patients whose imaging demonstrates the presence of a physiological response on either perfusion CT and/or ^{18}F-Misonidazole PET-CT will be classified as a responder whilst those that do not show a response on either imaging modality will be classed as a non-responder.

16.3 Randomisation

None planned.

17 STATISTICAL ANALYSIS PLAN

A detailed statistical analysis plan will be available from the time the first patient is recruited and will be finalised before any analysis is undertaken. The analysis plan will be written in accordance with the current OCTRU standard operating procedures and will be finalised and agreed by the trial statistician and the CI.

17.1 Inclusion in analysis

All patients enrolled in the study, will be accounted for and included in the analyses. Patients who have completed 56 days on trial or experience a DLT will be included in the dose escalation analysis. The number of patients who were not evaluable, who died or withdrew before treatment began will be recorded. The distribution of follow-up time will be described and the number of patients lost to follow-up will be given.

Variables will be analysed to determine whether the criteria for the study conduct are met. This will include a description of patients who did not meet all the eligibility criteria, an assessment of protocol violations, study drug accountability and other data that impact on the general conduct of the study.

Baseline characteristics will be summarised for all patients who commence BKM120. Patients who do not commence BKM120 or do not complete the required safety observations will be described separately.

Patients will be evaluable for safety and tolerability analysis from the time of their first treatment with BKM120 using NCIC CTCAE v 4.0 scoring and MedDRA coding. Treatment related toxicity will be tabulated by type and grade of toxicity.

Adverse events will be summarised by the number of patients experiencing each type of event. The grades and causality will be reported.

The functional imaging investigations will be used to assess whether BKM120 induces changes in tumour hypoxia and perfusion and to determine whether to open cohort 4. The criteria on which this decision will be based is summarised in Section 12.1.

17.2 Subgroup analysis

Nil planned.

17.3 Interim Analyses

Interim analyses will only be performed if requested by the Independent Early Phase Trial Oversight Committee (IEPTOC).

17.4 Procedures for reporting any deviation(s) from the original statistical plan

Any deviations from the original statistical plan will be described and justified in the final report.

17.5 Analysis of safety

Dose escalation phase
The variables that define the DLTs and safety variables will be summarized by descriptive statistics with patients grouped according to dose level received. Number (with percentages) of patient with and without DLT will also be presented according to dose level.

Expansion cohort and cohort 4
Safety and toxicity variables will be summarized by descriptive statistics to investigate the safety and feasibility of expanded cohort patients at MTD dose and cohort 4 patients at MTD dose for 3 weeks of BKM120.

17.6 Final analysis

The duration of the trial will depend upon whether 3 or 6 evaluable patients are required to complete each of cohorts 1 to 3, and whether there is a requirement to open cohort 4. Final analysis will be performed at least six weeks after the last patient in the trial has completed radiotherapy treatment. However, the analysis of escalation phase will be done and could be showed before expansion phase and/or cohort 4 analyses.

Expansion cohort and cohort 4
The variables related to the secondary endpoints of changes in ^{18}F-Misonidazole PET-CT and in blood flow will be summarized by descriptive statistics with patients grouped according to MTD cohort, expansion cohort and cohort 4. Number (with percentages) of patients ‘responder’ and ‘non-responder’ will also be presented according to the cohort of the patient.

18 TRIAL COMMITTEES

18.1 Trial Management Group (TMG)

The Chief Investigator will chair a TMG with responsibility for overseeing the successful conduct and publication of the trial in accordance with the protocol. The TMG will review safety and dose escalations with advice from the Independent Early Phase Trial Oversight Committee (see section 18.2 below). It will provide regular progress reports as required by the applicable steering committees and governance bodies. Members of the TMG are:

- Chief Investigator
- Clinical Trial Coordinator
- Trial management / QA
- Trial Statistician
- External Member

18.2 Data and Safety Monitoring

There is no Data and Safety Monitoring Committee. The IEPTOC will be in place to monitor the safety and progress of the trial.

18.3 Trial Steering Committee

The role of the Trial Steering Committee will be fulfilled by the IEPTOC.

19 DATA MANAGEMENT

19.1 Database considerations

Data management will be performed via a web-based, bespoke trial database (OpenClinica). OpenClinica is a dedicated and validated clinical trials database designed for electronic data capture. See: http://www.openclinica.org.

The Chief Investigator will act as Data Custodian for the trial.

19.2 Electronic case reports forms (eCRFs)

The Investigator and study site staff will ensure that data collected on each subject is recorded in the CRF as accurately and completely as possible. The CRFs will not contain any source data. All appropriate laboratory data, summary reports and Investigator observations will be transcribed into the CRFs from the relevant medical record(s). Please ensure that

- the relevant CRFs are completed and signed off by the Investigator for each study patient
- all CRF data are verifiable in the source documentation or the discrepancies must be explained.
- CRF sections are completed in a timely fashion, as close to the visit or event being recorded as possible.
- Data queries are resolved and documented by authorised study staff, giving a reason for the change or correction where appropriate.

The above considerations also apply to patients who are withdrawn early. If a patient withdraws from the study, the reason must be noted on the appropriate form and the patient must be followed-up as per protocol.

19.3 Accounting for missing, unused, or spurious data.

Missing data will be chased up and supplemented where possible after consultation with the investigator. The control of the correctness of the data is performed with ranking tests, validity tests and consistency checks. Unused data will be retained as for used data.

20 CLINICAL STUDY REPORT

All clinical data will be presented at the end of the study as data listings. These will be checked to confirm the lists accurately represents the data collected during the course of the study. The trial data will then be locked and a final data listing produced. The clinical study report will be based on the final data listings. The locked trial data may then be used for analysis and publication.

21 STUDY SITE MANAGEMENT

21.1 Study site responsibilities

The Principal Investigator (the PI or lead clinician for the study site) has overall responsibility for conduct of the study, but may delegate responsibility where appropriate to suitably experienced and trained members of the study site team. All members of the study site team must complete the delegation log provided prior to undertaking any study duties. The PI must counter sign and date each entry in a timely manner. Study team members must provide a current CV giving evidence of valid and up to date GCP training for inclusion in the study file. Copies of the delegation log and CVs will be provided to the trial office on request. Sites must report any unintended deviations/violations to OCTO according to the procedure outlined during site initiation training.
For further information on trial responsibilities, please refer to the ICH GCP guideline (E6). Copies can be obtained from www.ich.org

21.2 Study site set up and activation

A Principal Investigator should lead the study at the site, providing the local study office with all core documentation and attend 'Site Training Call' organised by the trial office before the site becomes activated (usually carried out as a telephone conference call or personal visit). The trial office will call to check that the site has all the required study information/documentation and is ready to recruit. The site will then be notified once they are activated on the BKM120 database and able to register.

21.3 Study documentation

The trial office will provide an Investigator File and Pharmacy File (where applicable) to each investigational site containing the documents needed to initiate and conduct the study. The trial office must review and approve any local changes made to any study documentation including patient information and consent forms prior to use. Additional documentation generated during the course of the trial, including relevant communications must be retained in the site files as necessary to reconstruct the conduct of the trial.

22 REGULATORY AND ETHICAL CONSIDERATIONS

The Sponsor and Investigators will ensure that this protocol will be conducted in compliance with the UK Clinical Trials Regulations[1], the principles of GCP and the applicable policies of the sponsoring organisation. Together, these implement the ethical principles of the Declaration of Helsinki (1996) and the regulatory requirements for clinical trials of an investigational medicinal product under the European Union Clinical Trials Directive.

22.1 Ethical conduct of the trial and ethics approval

The protocol, patient information sheet, consent form and any other information that will be presented to potential trial patients (e.g. advertisements or information that supports or supplements the informed consent) will be reviewed and approved by an appropriately constituted, independent Research Ethics Committee (REC).

22.2 Regulatory Authority approval

This study will be conducted under a CTA granted by the MHRA.

22.3 NHS Research Governance

Investigators are responsible for ensuring they obtain local Trust management agreement to conduct the trial in accordance with local arrangements and policies.

22.4 Protocol amendments

Amendments are changes made to the research following initial approval. A ‘substantial amendment’ is an amendment to the terms of the Responsible Authority application (if applicable), the REC application, or to the protocol or any other supporting documentation, that is likely to affect to a significant degree:

- the safety or physical or mental integrity of the subjects of the trial;
- the scientific value of the trial;
- the conduct or management of the trial; or
- the quality or safety of the investigational medicinal product(s) used in the trial.

Non-substantial amendments are those where the change(s) involve only minor logistical or administrative aspects of the study.

All amendments will be generated and managed according to the trial office standard operating procedures to ensure compliance with applicable regulation and other requirements. Written confirmation of all applicable REC, regulatory and local approvals must be in place prior to implementation by Investigators. The only exceptions are for changes necessary to eliminate an immediate hazard to study patients (see below).

It is the Investigator’s responsibility to update patients (or their authorised representatives, if applicable) whenever new information (in nature or severity) becomes available that might affect the patient’s willingness to continue in the trial. The Investigator must ensure this is documented in the patient’s medical notes and the patient is re-consented if appropriate.

22.5 Urgent safety measures

The sponsor or Investigator may take appropriate urgent safety measures to protect trial participants from any immediate hazard to their health or safety. Urgent safety measures may be taken without prior authorisation. The trial may continue with the urgent safety measures in place. **The Investigator must inform the trial office IMMEDIATELY if the study site initiates an urgent safety measure:**

Contact the BKM120 Trial Office on tel: 01865 617083 or fax: 01865 617010
or email: octo-BKM120@oncology.ox.ac.uk

The notification must include:

- Date of the urgent safety measure;
- Who took the decision; and
- Why the action was taken.

1 The Medicines for Human Use (Clinical Trials) Regulations (S.I. 2004/1031) and any subsequent amendments to it.

The Investigator will provide any other information that may be required to enable the trial office to report and manage the urgent safety measure in accordance with the current regulatory and ethical requirements for expedited reporting and close out.

22.6 Temporary halt

The sponsor and Investigators reserve the right to place recruitment to this protocol on hold for short periods for administrative reasons **or** to declare a temporary halt. A temporary halt is defined as a formal decision to:

- interrupt the treatment of subjects already in the trial for safety reasons;
- stop recruitment on safety grounds; or
- stop recruitment for any other reason(s) considered to meet the substantial amendment criteria, including possible impact on the feasibility of completing the trial in a timely manner.

The trial office will report the temporary halt via an expedited substantial amendment procedure. The trial may not restart after a temporary halt until a further substantial amendment to re-open is in place. If it is decided not to restart the trial this will be reported as an early termination.

22.7 Serious Breaches

The Medicines for Human Use (Clinical Trials) Regulations require the Sponsor to notify any "serious breaches" to the MHRA within 7 days of the sponsor becoming aware of the breach. A serious breach is defined as “A breach of GCP or the trial protocol which is likely to effect to a significant degree:

- the safety or physical or mental integrity of the subjects of the trial; or
- the scientific value of the trial

Investigators must notify the trials office at once if any serious breach of GCP is suspected.

22.8 Progress, end of study and other reports

This protocol will comply with all current applicable Regulatory Authority, Research Ethics Committee and Sponsor requirements for the provision of periodic study safety and progress reports. Any additional reports will be provided on request. Reporting will be managed by the trials office according to internal Standard Operating Procedures.

Trial reports will be copied to all Principal Investigators as appropriate. Study sites are responsible for forwarding these reports to their local Trust as required.

23 EXPENSES AND BENEFITS

Reasonable travel expenses for any visits additional to normal care will be reimbursed on production of receipts or a mileage allowance provided as appropriate.

24 QUALITY ASSURANCE

24.1 Risk assessment and site monitoring

A risk assessment based monitoring plan will be prepared before the study opens. The risk assessment will be repeated if necessary in the light of changes while the study is ongoing or in response to monitoring reports. The Monitoring plan will be amended as appropriate.

Regular monitoring will be performed according to the plan. Data will be evaluated for compliance with the protocol and accuracy in relation to source documents. Monitoring visits reports will be sent to the site in a timely fashion.

24.2 Central monitoring

The site will be monitored centrally by checking incoming forms for compliance with the protocol, data consistency, missing data and timing or the database which validates data against set criteria and ranges. All changes to data that could influence the outcome will be queried with and approved by the study site in a timely manner. For all other data, where there is no doubt about the source of any errors, clear changes to data will be made internally by OCTO staff without referring back to the study site. Study staff will be in regular contact with site personnel (by phone/fax/email/letter) to check on progress and deal with any queries that they may have including those arising from queries raised by the trials office.

24.3 Audit and Regulatory Inspection

All aspects of the study conduct may be subject to internal or external quality assurance audit to ensure compliance with the protocol, GCP requirements and other applicable regulation or standards. It may also be subject to a regulatory inspection. Such audits or inspections may occur at any time during or after the completion of the study. Investigators and their host Institution(s) should understand that it is necessary to allow auditors/inspectors direct access to all relevant documents, study facilities and to allocate their time and the time of their staff to facilitate the audit or inspection visit. Anyone receiving notification of a Regulatory Inspection that will (or is likely to) involve this trial must inform the trial office without delay.

25 RECORDS RETENTION & ARCHIVING

During the clinical trial and after trial closure the Investigator must maintain adequate and accurate records to enable the conduct of a clinical trial and the quality of the research data to be evaluated and verified. All essential documents (as detailed in OCTO's SOP) must be stored in such a way that ensures that they are readily available, upon request for the minimum period required by national legislation or for longer if needed. The medical files of trial subjects shall be retained in accordance with national legislation and in accordance with the host institution policy.

Retention and storage of laboratory records for clinical trial samples must also follow these guidelines.
Retention and storage of central laboratory records supporting PK or PD endpoints and the disposition of samples donated via the trial must also comply with applicable legislation and Sponsor requirements.

It is the University of Oxford's policy to store data for a minimum of 5 years. Investigators may not archive or destroy study essential documents or samples without written instruction from the trial office.

26 PATIENT CONFIDENTIALITY

The personal data recorded on all documents will be regarded as confidential, and to preserve each patient's anonymity, only their initials and date of birth will be recorded on the CRFs.

Medical source data or Scans acquired and exported for research purposes will be linked anonymised as per Departmental Protocol so that the patient cannot be identified from them.

The Investigator site must maintain the patient's anonymity in all communications and reports related to the research. The Investigator site team must keep a separate log of enrolled patients' personal identification details as necessary to enable them to be tracked. These documents must be retained securely, in strict confidence. They form part of the Investigator Site File and are not to be released externally.

27 STUDY FUNDING

This study is funded by the Oxford Cancer Imaging Centre, Cancer Research UK and the Experimental Cancer Medicine Centre network. Novartis are providing BKM120 and providing funding for packaging and distribution.

28 SPONSORSHIP AND INDEMNITY

28.1 Sponsorship

The Sponsor will provide written confirmation of Sponsorship and authorise the trial commencement once satisfied that all arrangements and approvals for the proper conduct of the trial are in place. A separate study delegation agreement, setting out the responsibilities of the Chief Investigator and Sponsor will be put in place between the parties.

28.2 Indemnity

Compensation for Harm
Arrangements for NEGLIGENT harm
Indemnity and/or compensation for negligent harm arising specifically from accidental injury for which the University is legally liable as the Research Sponsor will be covered by the University of Oxford. The NHS will owe a duty of care to those undergoing clinical treatment, with Trust Indemnity available through the NHS Litigation Authority Scheme.

Arrangements for NON-NEGLIGENT harm
Indemnity and/or compensation for harm arising specifically from an accidental injury and occurring as a consequence of the research subjects' participation in the trial for which the University is the research Sponsor may be covered by the University of Oxford.

28.3 Contracts/Agreements

This trial is subject to the Sponsors policy requiring that written contracts/agreements are agreed formally by the participating bodies as appropriate. A Clinical Trial Agreement (CTA) will be placed between the Sponsor and participating NHS Trust(s) prior to site activation.
The Sponsor will also set up written agreements with any other external third parties involved in the conduct of the trial as appropriate.

29 PUBLICATION POLICY

The Chief and Co-Investigators (the investigators) will retain ownership of all data arising from this trial. The intention is to publish this research in a specialist peer reviewed scientific journal on completion of the study. The results may also be presented at scientific meetings and/or used for a thesis. The Investigators will be involved in reviewing drafts of the manuscripts, abstracts, press releases and any other publications arising from the trial and retain final editorial control.

Authors will acknowledge that the study was sponsored by and performed with the support of the Sponsor, and with funding support from Cancer Research UK, the Oxford Cancer Imaging Centre and the Oxford ECMC. Novartis has a right to review each publication and presentation (including, but not limited to, full papers, abstracts, poster presentations and oral presentations), prior to submission. Also, trial reports, entries on clinical trial databases, publications including abstracts and presentations shall acknowledge Novartis' support.

30 REFERENCES

1. Bendell JC, Rodon J, Burris HA, de Jonge M, Verweij J, Birle D, et al. Phase I, Dose-Escalation Study of BKM120, an Oral Pan-Class I PI3K Inhibitor, in Patients With Advanced Solid Tumors. J Clin Oncol. 2011.

2. Hugonnet F, Fournier L, Medioni J, Smadja C, Hindie E, Huchet V, et al. Metastatic renal cell carcinoma: relationship between initial metastasis hypoxia, change after 1 month's sunitinib, and therapeutic response: an 18F-fluoromisonidazole PET/CT study. J Nucl Med. 2011;52:1048-55.

3. Fraioli F, Anzidei M, Zaccagna F, Mennini ML, Serra G, Gori B, et al. Whole-tumor perfusion CT in patients with advanced lung adenocarcinoma treated with conventional and antiangiogenetic chemotherapy: initial experience. Radiology. 2011;259:574-82.

4. Maity A, Bernhard EJ. Modulating tumor vasculature through signaling inhibition to improve cytotoxic therapy. Cancer research. 2010;70:2141-5.

5. Begg AC, Stewart FA, Vens C. Strategies to improve radiotherapy with targeted drugs. Nat Rev Cancer. 2011;11:239-53.

6. McKenna WG, Muschel RJ, Gupta AK, Hahn SM, Bernhard EJ. The RAS signal transduction pathway and its role in radiation sensitivity. Oncogene. 2003;22:5866-75.

7. Gupta AK, Soto DE, Feldman MD, Goldsmith JD, Mick R, Hahn SM, et al. Signaling pathways in NSCLC as a predictor of outcome and response to therapy. Lung. 2004;182:151-62.

8. Vaupel P. Tumor microenvironmental physiology and its implications for radiation oncology. Semin Radiat Oncol. 2004;14:198-206.

9. Ray GM. Antiretroviral and statin drug-drug interactions. Cardiol Rev. 2009;17:44-7.

10. Sahani DV, Kalva SP, Hamberg LM, Hahn PF, Willett CG, Saini S, et al. Assessing tumor perfusion and treatment response in rectal cancer with multisection CT: initial observations. Radiology. 2005;234:785-92.

11. Okamoto S, Shiga T, Yasuda K, Ito YM, Magota K, Kasai K, Kuge Y, Shirato H, Tamaki N. High Reproductibility of Tumour Hypoxia Evaluated by 18F-Fluoromisonidazole PET for Head and Neck Cancer. J Nucl Med. 2013; 54:201-207

12. Copay AG, Subach BR, Glassman SD, Polly DW Jr, Schuler TC. Understanding the minimum clinically important difference: a review of concepts and methods. The Spine Journal. 2007;7: 541-546.

APPENDIX 1: ECOG PERFORMANCE SCALE

Activity Performance Description	Score
Fully active, able to carry out all on all pre-disease performance without restriction.	0
Restricted in physically strenuous activity but ambulatory and able to carry out work of a light or sedentary nature, e.g. light housework, office work.	1
Ambulatory and capable of all self-care, but unable to carry out any work activities. Up and about more than 50% of waking hours.	2
Capable of only limited self-care. Confined to bed or chair more than 50% of waking hours.	3
Completely disabled. Cannot carry out any self-care. Totally confined to bed or chair.	4

APPENDIX 2: LIST OF CYP450 SUBSTRATES TO BE USED WITH CAUTION

CYP2C8	CYP2C9	CYP2C19	CYP3A4,5,7**	
amodiaquine	celecoxib	amitriptyline	adinazolam	fentanyl[2]
cerivastatin	diclofenac	citalopram	alfentanil[1,2]	flunitrazepam
repaglinide	flurbiprofen	clobazam	alphadihydroergocryptine[1]	fluticasone[1]
R-ibuprofen	fluvastatin	clomipramine	alprazolam	lovastatin[1]
rosiglitazone	glibenclamide (glyburide)	clopidogrel	amlodipine	maraviroc[1]
	gliclazide	diazepam	aripiprazole	midazolam[1]
	glimepiride	fluoxetine	atorvastatin	nifedipine
	glipizide	imipramine	brotizolam[1]	nisoldipine
	indomethacin	lansoprazole	budesonide[1]	nitrendipine
	irbesartan	moclobemide	buspirone[1]	perospirone[1]
	ketobemidone	omeprazole	cerivastatin	quinine
	lornoxicam	pantoprazole	chlorpheniramine	sildenafil[1]
	losartan	progesterone	cyclosporine[2]	simvastatin[1]
	meloxicam	propranolol	darifenacin[1]	sirolimus[1,2]
	naproxen	quazepam	diazepam	tipranavir[1]
	nateglinide	rabeprazole	diergotamine[2]	trazodone
	piroxicam	sertraline	ebastine[1]	triazolam[1]
	S-ibuprofen	S-mephenytoin	eletriptan[1]	
	sulfamethoxazole		eplerenone[1]	
	tenoxicam		ergotamine[2]	
	tolbutamide		estazolam	
	torasemide		everolimus[1]	
	valdecoxib		felodipine[1]	

APPENDIX 3: LIST OF PROHIBITED QT PROLONGING DRUGS

Drug	QT risk(*)	Comment
Amiodarone	Known risk for TdP	Females>Males, TdP risk regarded as low
Arsenic trioxide	Known risk for TdP	
Astemizole	Known risk for TdP	No longer available in US.<br>CYP3A4 substrate with narrow therapeutic index.
Bepridil	Known risk for TdP	Females>Males
Chloroquine	Known risk for TdP	
Chlorpromazine	Known risk for TdP	
Cisapride	Known risk for TdP	Restricted availability; Females>Males.<br>CYP3A substrate with narrow therapeutic index.
Disopyramide	Known risk for TdP	Females>Males
Dofetilide	Known risk for TdP	
Domperidone	Known risk for TdP	Not available in the US.
Droperidol	Known risk for TdP	
Halofantrine	Known risk for TdP	Females>Males
Haloperidol	Known risk for TdP	When given intravenously or at higher-than- recommended doses, risk of sudden death, QT prolongation and torsades increases.
Ibutilide	Known risk for TdP	Females>Males
Levomethadyl	Known risk for TdP	Sensitive CYP3A substrate
Mesoridazine	Known risk for TdP	
Methadone	Known risk for TdP	Females>Males
Pentamidine	Known risk for TdP	Females>Males
Pimozide	Known risk for TdP	Females>Males. Sensitive CYP3A substrate with narrow therapeutic index
Probucol	Known risk for TdP	No longer available in U.S.
Procainamide	Known risk for TdP	
Quetiapine	Possible risk for TdP	Sensitive CYP3A substrate
Quinidine	Known risk for TdP	Females>Males. Sensitive CYP3A substrate
Sotalol	Known risk for TdP	Females>Males
Sparfloxacin	Known risk for TdP	
Tacrolimus	Possible risk for TdP	Sensitive CYP3A substrate with narrow therapeutic index
Terfenadine	Known risk for TdP	No longer available in U.S. Sensitive CYP3A substrate with narrow therapeutic index
Thioridazine	Known risk for TdP	
Vardenafil	Possible risk for TdP	Sensitive CYP3A substrate
(*) Classification according to the Qtdrugs.org Advisory Board of the Arizona CERT<br>Sensitive substrates: Drugs whose plasma AUC values have been shown to increase 5-fold or higher when co-administered with a potent inhibitor of the respective enzyme.		

APPENDIX 4: LIST OF QT PROLONGING DRUGS TO BE USED WITH CAUTION

1. Drug	2. QT risk (*)
Alfuzosin	possible risk for Torsades de Pointes
Amantadine	possible risk for Torsades de Pointes
Amitriptyline	conditional risk for Torsades de Pointes
Azithromycin	possible risk for Torsades de Pointes
Chloral hydrate	possible risk for Torsades de Pointes
Citalopram	conditional risk for Torsades de Pointes
Clomipramine	conditional risk for Torsades de Pointes
Clozapine	possible risk for Torsades de Pointes
Desipramine	conditional risk for Torsades de Pointes

1. Drug	2. QT risk (*)
Diphenhydramine	conditional risk for Torsades de Pointes
Dolasetron	possible risk for Torsades de Pointes
Doxepin	conditional risk for Torsades de Pointes
Dronedarone	possible risk for Torsades de Pointes
Felbamate	possible risk for Torsades de Pointes
Flecainide	possible risk for Torsades de Pointes
Fluoxetine	conditional risk for Torsades de Pointes
Foscarnet	possible risk for Torsades de Pointes
Fosphenytoin	possible risk for Torsades de Pointes
Galantamine	conditional risk for Torsades de Pointes
Gatifloxacin	possible risk for Torsades de Pointes
Gemifloxacin	possible risk for Torsades de Pointes
Granisetron	possible risk for Torsades de Pointes
Imipramine	conditional risk for Torsades de Pointes
Indapamide	possible risk for Torsades de Pointes
Isradipine	possible risk for Torsades de Pointes
Levofloxacin	possible risk for Torsades de Pointes
Lithium	possible risk for Torsades de Pointes
Mexiletine	conditional risk for Torsades de Pointes
Moexipril/HCTZ	possible risk for Torsades de Pointes
Moxifloxacin	possible risk for Torsades de Pointes
Nicardipine	possible risk for Torsades de Pointes
Nortriptyline	conditional risk for Torsades de Pointes
Octreotide	possible risk for Torsades de Pointes
Ofloxacin	possible risk for Torsades de Pointes
Ondansetron	possible risk for Torsades de Pointes
Oxytocin	possible risk for Torsades de Pointes
Paliperidone	possible risk for Torsades de Pointes
Paroxetine	conditional risk for Torsades de Pointes
Perflutren lipid microspheres	possible risk for Torsades de Pointes
Protriptyline	conditional risk for Torsades de Pointes
Ranolazine	possible risk for Torsades de Pointes
Risperidone	possible risk for Torsades de Pointes
Roxithromycin*	possible risk for Torsades de Pointes
Sertindole	possible risk for Torsades de Pointes
Sertraline	conditional risk for Torsades de Pointes
Solifenacin	conditional risk for Torsades de Pointes
Tizanidine	possible risk for Torsades de Pointes
Trazodone	conditional risk for Torsades de Pointes
Trimethoprim-Sulfa	conditional risk for Torsades de Pointes
Trimipramine	conditional risk for Torsades de Pointes
Venlafaxine	possible risk for Torsades de Pointes
Ziprasidone	possible risk for Torsades de Pointes

APPENDIX 5: LIST OF PROHIBITED CYP3A INHIBITORS (STRONG AND MODERATE) AND INDUCERS

**Strong CYP3A4,5,7 inhibitors**	**Moderate CYP3A4,5,7 inhibitors**	**CYP3A4 inducers**
clarithromycin	aprepitant	barbiturates
conivaptan	atazanavir	carbamazepine
grapefruit juice	cimetidine	efavirenz

**Strong CYP3A4,5,7 inhibitors**	**Moderate CYP3A4,5,7 inhibitors**	**CYP3A4 inducers**
indinavir	ciprofloxacin	modafenil
itraconazole	darunavir	nevirapine
ketoconazole	diltiazem	oxcarbazepine
lopinavir	erythromycin	phenobarbital
mibefradil	fluconazole	phenytoin
nefazodone	tofisopam	pioglitazone
nelfinavir	verapamil	rifabutin
posaconazole	amprenavir	rifampin
ritonavir	fosamprenavir	St. John's wort
saquinavir		topiramate
telithromycin		troglitazone
troleandomycin		
voriconazole		

APPENDIX 6: PATIENT SELF REPORTED MOOD QUESTIONNAIRES

GAD-7 anxiety scale

Over the last 2 weeks, how often have you been bothered by the following problems? (Use "✔" to indicate your answer)	Not at all	Several Days	More than half of the days	Nearly every day
1. Feeling nervous, anxious or on edge	0	1	2	3
2. Not being able to stop or control worrying	0	1	2	3
3. Worrying too much about different things	0	1	2	3
4. Trouble relaxing	0	1	2	3
5. Being so restless that it is hard to sit still	0	1	2	3
6. Becoming easily annoyed or irritable	0	1	2	3
7. Feeling afraid as if something awful might happen	0	1	2	3
Column totals:	______	+ ______	+ ______	+ ______

= Total Score ______

PHQ9 depression scale

Over the last 2 weeks, how often have you been bothered by any of the following problems?<br><br>(Use "✔" to indicate your answer)	Not at all	Several days	More than half the days	Nearly every day
1. Little interest or pleasure in doing things	0	1	2	3
2. Feeling down, depressed, or hopeless	0	1	2	3
3. Trouble falling or staying asleep, or sleeping too much	0	1	2	3
4. Feeling tired or having little energy	0	1	2	3
5. Poor appetite or overeating	0	1	2	3
6. Feeling bad about yourself - or that you are a failure or have let yourself or your family down	0	1	2	3
7. Trouble concentrating on things, such as reading the newspaper or watching television	0	1	2	3
8. Moving or speaking so slowly that other people Could have noticed? Or the opposite - being so fidgety or restless that you have been moving around a lot more than usual	0	1	2	3
9. Thoughts that you would be better off dead or of hurting yourself in some way	0	1	2	3
Column totals:	_____+	_____+	_____+	_____+
	= Total Score			

If you checked off any problems, how difficult have these problems made it for you to do your work, take care of things at home, or get along with other people?

Not difficult at all	Somewhat difficult	Very difficult	Extremely difficult

APPENDIX 7: AMENDMENT HISTORY

Amendment No.	Protocol Version No.	Date issued	Author(s) of changes	Details of Changes made
N/A	V2.0	24 Dec 2012	Linda Collins	N/A Protocol V2.0 was the protocol initially approved for use in the trial
001	V3.0	22 Mar 2013	Linda Collins	Change to scan procedure
003	V4.0	16 Sep 2013	Linda Collins	Change to scan procedure
006	V5.0	07 Jul 2014	Naomi McGregor	DLT definitions
010	V6.0	24 Mar 2016	Naomi McGregor	Guidance on management of liver and mood alteration toxicity
011	V7.0	12 Jan 2016	Stasya Ng	Eligibility criteria (ECOG performance score)
